# Genome Sequence of *Candidatus* Nitrososphaera evergladensis from Group I.1b Enriched from Everglades Soil Reveals Novel Genomic Features of the Ammonia-Oxidizing Archaea

**DOI:** 10.1371/journal.pone.0101648

**Published:** 2014-07-07

**Authors:** Kateryna V. Zhalnina, Raquel Dias, Michael T. Leonard, Patricia Dorr de Quadros, Flavio A. O. Camargo, Jennifer C. Drew, William G. Farmerie, Samira H. Daroub, Eric W. Triplett

**Affiliations:** 1 Microbiology and Cell Science Department, Institute of Food and Agricultural Sciences, University of Florida, Gainesville, Florida, United States of America; 2 Soil Science Department, Federal Unviersity of Rio Grande do Sul, Porto Alegre, RS, Brazil; 3 Genome Sequencing Services Laboratory, Interdisciplinary Center for Biotechnology Research, University of Florida, Gainesville, Florida, United States of America; 4 Everglades Research and Education Center, University of Florida, Belle Glade, Florida, United States of America; Auburn University, United States of America

## Abstract

The activity of ammonia-oxidizing archaea (AOA) leads to the loss of nitrogen from soil, pollution of water sources and elevated emissions of greenhouse gas. To date, eight AOA genomes are available in the public databases, seven are from the group I.1a of the Thaumarchaeota and only one is from the group I.1b, isolated from hot springs. Many soils are dominated by AOA from the group I.1b, but the genomes of soil representatives of this group have not been sequenced and functionally characterized. The lack of knowledge of metabolic pathways of soil AOA presents a critical gap in understanding their role in biogeochemical cycles. Here, we describe the first complete genome of soil archaeon *Candidatus* Nitrososphaera evergladensis, which has been reconstructed from metagenomic sequencing of a highly enriched culture obtained from an agricultural soil. The AOA enrichment was sequenced with the high throughput next generation sequencing platforms from Pacific Biosciences and Ion Torrent. The *de novo* assembly of sequences resulted in one 2.95 Mb contig. Annotation of the reconstructed genome revealed many similarities of the basic metabolism with the rest of sequenced AOA. *Ca*. N. evergladensis belongs to the group I.1b and shares only 40% of whole-genome homology with the closest sequenced relative *Ca*. N. gargensis. Detailed analysis of the genome revealed coding sequences that were completely absent from the group I.1a. These unique sequences code for proteins involved in control of DNA integrity, transporters, two-component systems and versatile CRISPR defense system. Notably, genomes from the group I.1b have more gene duplications compared to the genomes from the group I.1a. We suggest that the presence of these unique genes and gene duplications may be associated with the environmental versatility of this group.

## Introduction

The ammonia-oxidizing archaea (AOA) are an abundant group of nitrifiers that plays important environmental roles in the open oceans, soils, the arctic, hot springs and marine sponges [1]–[8]. AOA oxidize ammonia (NH_3_) to nitrite (NO_2_^−^) with further oxidation to nitrate (NO_3_^−^) by nitrite-oxidizing bacteria [9], [10]. In soils, nitrification can increase mobility of inorganic N, hence it may cause NO_3_^−^ leaching from soils, pollution of ground and surface waters, and an increased cost of applied N fertilizers in agricultural areas [11]–[13].

Another possible negative consequence of AOA activity in marine environments and soil, particularly in agricultural areas, is the increased pollution of the atmosphere by nitrous oxide (N_2_O). Nitrous oxide is one of the most stable greenhouse gases, and agricultural soil management is the largest source of N_2_O emissions in the United States (69% of total U.S. N_2_O emissions) [14]. Several studies demonstrate that AOA produce N_2_O [15]–[18]. However, the underlying pathways for biogeoproduction of N_2_O remain unknown.

AOA are difficult to culture. Only a few AOA have been cultured and sequenced from either pure or enrichment cultures [2]–[6], [19], [20]. When AOA were first discovered, major AOA groups (I.1a, I.1b and Hot spring cluster) were proposed by 16S rRNA gene and *amoA* gene identities [10]. Group I.1b (or *Nitrososphaera* cluster) is mostly represented by AOA from soil and some other habitats, including hot springs, freshwater, and freshwater sediments [8], [21]–[23]. Group I.1a (or *Nitrosopumilus* cluster) is mainly represented by marine archaea. However it has also been found in other environments including soil, hot springs, and freshwater [1]–[6], [19], [22], [24]. Seven genome sequences from group I.1a (*Nitrosopumilus mritimus*, *Candidatus* Nitrosopumilus sediminis, *Candidatus* Nitrosopumilus salaria, *Candidatus* Nitrosoarchaeum limnia, *Candidatus* Nitrosopumilus koreensis, *Cenarchaeum symbiosum*, and *Candidatus* Nitrosotenuis uzonensis) and only one from group I.1b (*Candidatus* Nitrososphaera gargensis) are available in public databases.

The lack of genomic information limits our understanding of the physiology and biochemistry of AOA, particularly Thaumarchaeota from group I.1b. Furthermore, the recently sequenced genome of moderate thermophile *Ca*. Nitrososphaera gargensis from the group I.1b revealed some differences between I.1b and I.1a groups of Thaumarchaeota [20]. For example, the genome size of *Ca*. Nitrososphaera gargensis is much bigger than other sequenced genomes from group I.1a. Additionally, the sequence analysis indicated higher G+C content, more thermosome genes, and a different chemical structure of membrane lipids [20],[25]. However, *Ca*. Nitrososphaera gargensis was isolated from hot springs, and it is unknown whether these features are specific only to thermophilic archaeon, or if mesophilic AOA, widely distributed in soils, from group I.1b also share these features.

From a previous study we found that AOA closely related to *Nitrososphaera* genus are highly abundant in the Everglades Agricultural Area, and their abundance significantly increases with agricultural management [26]. In this paper, we present (a) the preparation of an enriched culture of AOA from an Everglades histosol soil; (b) the sequencing and genome reconstruction of the first mesophilic AOA from the group I.1b enriched from the soil; (c) the genome annotation and analysis of main physiological features; and (d) the major metabolic differences between group I.1b and group I.1a.

For the first time, we report genome analysis of AOA group I.1b isolated from soil. This genome provides insight into genomic features present in *Ca*. N. evergladensis but not in other sequenced AOA. The genome analysis reveals features that distinguish AOA from I.1b and I.1a groups. This study provides important insight to guide our understanding of the role of AOA in terrestrial and marine environments.

## Results and Discussion

### Preparation of ammonia-oxidizing enrichment culture

An AOA enrichment culture was prepared from soil collected from the Everglades Agricultural Area using AOA medium and culture conditions described previously [21]. However, the addition of antibiotics to the enrichment culture did not result in pure culture as some other microorganisms remained. Preliminary genetic analysis of the AOA enrichment was performed by 16S rRNA amplification and Sanger sequencing of the clone library. Approximately 50% of all 16S rRNA clones were assigned to *Nitrososphaera* genus.

Enrichment was tested for the presence of ammonia-oxidizing bacteria (AOB) by PCR-amplification of the bacterial *amoA* genes. No amplification of the bacterial *amoA* was observed. In addition, sequence search of the bacterial *amoA* and 16S rRNA of AOB in the metagenomic sequences of the enrichment was performed against a customized database of bacterial *amoA* sequences and the reference Ribosomal Database Project (RDP) 16S SSU rRNA database [27]. This search did not reveal either bacterial *amoA* or known 16S rRNA genes affiliated with AOB. Further metagenomic analysis of the AOA enrichment showed that all present archaeal *amoA* (12 gene copies) corresponded to *amoA* from the *Ca*. N. evergladensis genome (NTE_00961) at the level of amino-acid identity 99.1–100%. Consistent with these results, all identified archaeal 16S rRNA (9 gene copies) in the enrichment displayed 99.1–100% of nucleotide identity with Ca. N. evergladensis 16S rRNA (NTE_02406).

**Figure 1:**
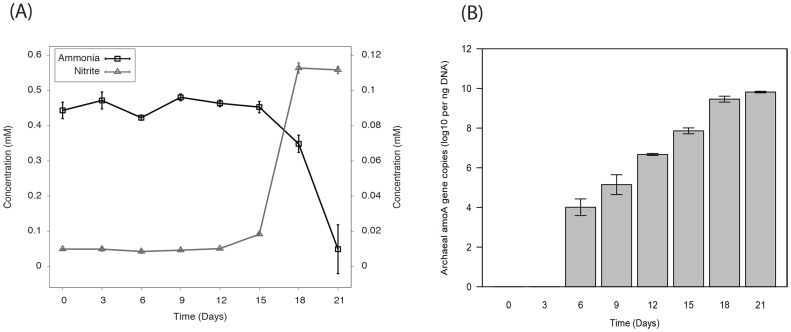
Correlation of ammonia oxidation with the growth of *Ca*. Nitrososphaera evergladensis enrichment culture incubated for 21 days. (A) Concentrations of ammonia and nitrite were determined spectrophotometrically. (B) Archaeal *amoA* gene copies were measured by quantitative PCR.

Ammonia consumption and nitrite (NO_2_^−^) production, as well as archaeal *amoA* gene copy number, were measured every three days after inoculation (Figure 1A and 1B). Ammonia was converted to NO_2_^−^ over a period of about 21 days (Figure 1A). Simultaneous oxidation of NH_3_ and production of NO_2_^−^ was accompanied by the increase of archaeal *amoA* gene copy number (Figure 1B).

### Gene prediction and annotation

The genome of the enriched AOA was reconstructed from assembled reads generated using data from the Pacific Biosciences (PacBio) platform (Figure S1A in File S1). Genome assembly was verified by PCR of selected regions in the assembled genome and by alignment of the genome with the contigs obtained from sequencing results from an Ion Torrent platform (Figure S1B in File S1). The 2.95 Mb genome sequence included 3555 genes, 50% G+C content, and 43 RNA genes. Over eighty percent (83.6%) of the assembled bases were predicted to code for proteins. Only 52% of protein coding sequences had functional assignment. Moreover, 60.6% of identified genes were in paralog clusters.

### Phylogeny and general genome features of *Ca*. N. evergladensis

**Figure 2:**
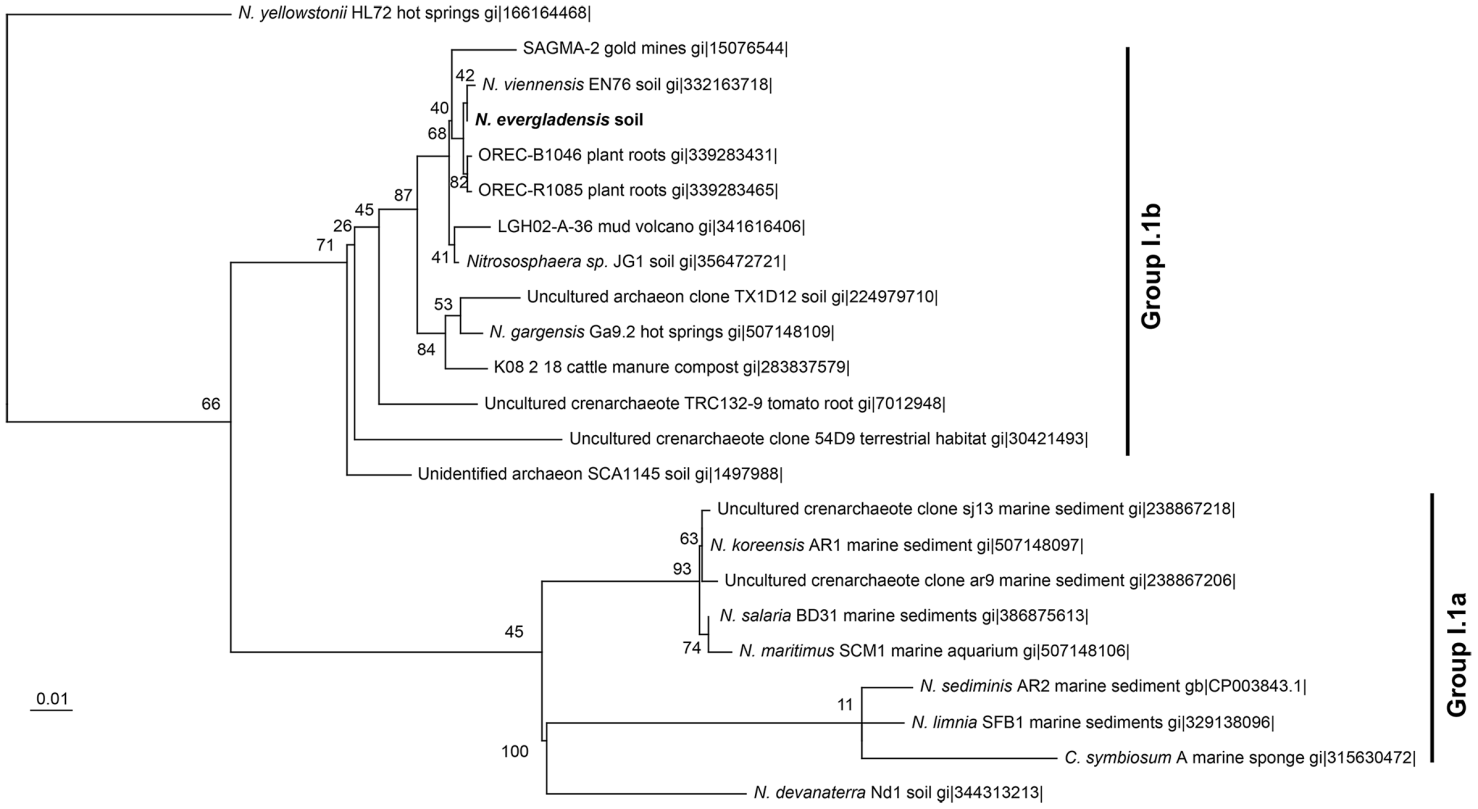
A phylogenetic tree of ammonia-oxidizing archaea 16S rRNA gene sequences (about 1.4 kb). 23 16S rRNA sequences of AOA were randomly selected from the National Center for Biotechnology Information databases. Conservative sites (1.08 kb) were selected using Gblocks. The branching patterns in the maximum-likelihood tree are denoted by their respective bootstrap values (1000 iterations).

**Figure 3:**
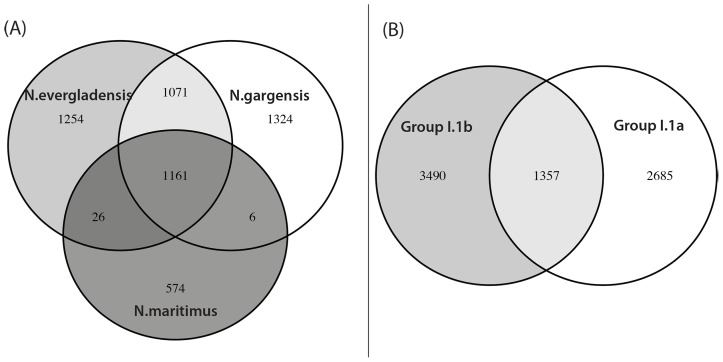
Comparison of protein coding sequences (CDS) of *Ca*. Nitrososphaera evergladensis with CDS of other ammonia-oxidizing archaea. (A) CDS of *Ca*. Nitrososphaera evergladensis were compared to CDS of *Ca*. N. gargensis. (B) CDS of the group I.1a (*N. maritimus*, *Ca*. N. sediminis, *C. symbiosum*, *Ca*. N. limnia, *Ca*. N. koreensis) were compared to CDS of the group I.1b (*Ca*. N. evergladensis and *Ca*. N. gargensis). Overlapping regions represent CDS with amino acid sequence identity 35% and higher.

**Figure 4:**
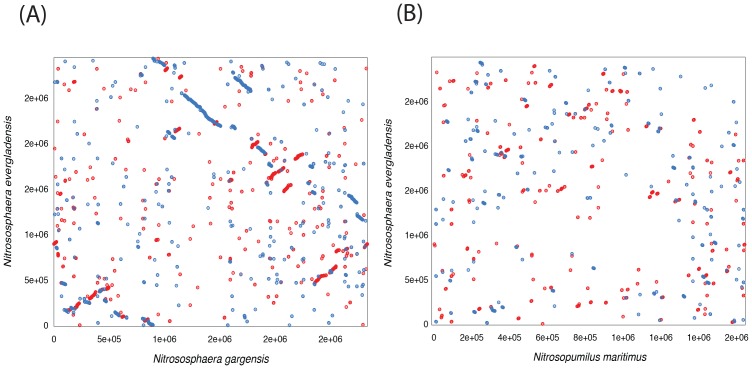
Genome synteny alignments of *Ca*. N. evergladensis, (A) *Ca*. N. gargensis and (B) *N. maritimus*. Axes X and Y represent topology of coding sequences in the comparing genomes. Entire genomes were compared by MUMmer 3.0 package using Promer tool [114]. Each dot represents a match of at least six amino acids from compared genomes. Forward matching amino acid sequences are plotted as red lines/dots while reverse are plotted as blue lines/dots. A line of dots with slope  = 1 represents an undisturbed segment of conservation between the two sequences, while a line of slope  = −1 represents an inverted segment of conservation between the two sequences.

Based on 16S rRNA and *amoA* classification, the mesophilic AOA *Ca*. N. evergladensis is phylogenetically affiliated with Thaumarchaeota from group I.1b (Figure 2, Figure S2 in File S1). The closest cultured relatives from the group I.1b are *Ca*. N. gargensis, *N. viennensis*, and *Nitrososphaera sp*. JG1 (Figure 2). *Ca*. N. evergladensis shares 97% and 85% 16S rRNA identity with *Ca*. N. gargensis and the AOA from group I.1a, respectively (Table A in File S1). Nucleotide identity of *amoA* genes were less conserved. *Ca*. N. evergladensis *amoA* was 87% and 71–74% identical to *Ca*. N. gargensis and group I.1a, respectively. AOA from the group I.1b have larger genomes and almost twice the number of protein coding sequences (CDS) compared to the group I.1a (Table A in File S1). Sixty-four percent of CDS from the *Ca*. N. evergladensis genome share 35% identity with *Ca*. N. gargensis and less than 34% of CDS were found in common with *N. maritimus* (Figure 3A). Overall, I.1a and I.1b groups shared about 30% CDS (Figure 3B). Whole-genome alignment of *Ca*. N. evergladensis to *Ca*. N. gargensis revealed 40% of conserved sites between two genomes. *Ca*. N. evergladensis shared a much smaller degree of genome synteny with *N. maritimus* than *Ca*. N gargensis (Figures 4A, 4B). An average nucleotide identity of 82.9% between both *Nitrososphaera* genomes confirmed that both genomes represent different species.

### Carbon metabolism

The *Ca*. N. evergladensis genome codes for the key enzymes of a 3-hydroxypropionate/4-hydroxybutyrate pathway of CO_2_ fixation, enzymes for forward and reverse tricarboxylic acid cycle (TCA) cycle, gluconeogenesis, a modified glycolytic pathway, and hexose monophosphate pathway (Table S1 in File S2, Figures S3, S4, S5, S6 in File S1).

#### 3-hydroxypropionate/4-hydroxybutyrate carbon fixation pathway

*Ca*. N. evergladensis, much like all known chemolithotrophic AOA, is predicted to fix inorganic carbon via a modified 3-hydroxypropionate/4-hydroxybutyrate cycle [3]. Despite the fact that key enzymes for this pathway were found in other sequenced AOA genomes, some steps of the AOA 3-hydroxypropionate/4-hydroxybutyrate cycle remain undescribed [1], [2], [20], [28]. Genes for the key enzymes for this pathway are found in the *Ca*. N. evergladensis genome (Figure S3 in File S1, Table S1 in File S2). These genes include alpha and beta subunits of acetyl-CoA carboxylase, acetyl/propionyl-CoA carboxylase, methylmalonyl-CoA epimerase and two domains of mutase. Also, biotin-(acetyl-CoA-carboxylase) ligase, which is responsible for assembly of carboxylase subunits, was identified in *Ca*. N. evergladensis. Candidates for missing enzymes that catalyze reactions of malonyl-CoA to propionyl-CoA were suggested by functional similarity and gene clustering (Table S1 in File S2).

The Thaumarchaeota were recently shown to possess a very efficient, aerobic pathway for CO_2_ fixation that differs from that found in the Crenarchaeota [29]. The *Ca*. N. evergladensis genome has all eleven of the genes identified to date in this pathway [29].

#### Tricarboxylic acid cycle

The reductive tricarboxylic acid cycle (TCA) is another potential pathway through which AOA may fix CO_2_ autotrophically [30]. Enzymes for both oxidative and reductive TCA cycles are predicted for *Ca*. N. evergladensis (Figure S4 in File S1, Table S1 in File S2). Two genes coding for the two subunits of 2-oxoglutarate oxidoreductase, proposed to catalyze interconversion of a-ketoglutarate and succinyl-CoA in the TCA cycle of the hyperthermophilic crenarchaeote *Thermoproteus tenax* [31], [32], were found adjacent to the gene coding for aconitase. Gene homologs for four subunits of the reversible succinate dehydrogenase/fumarate reductase were also detected in *Ca*. N. evergladensis. By contrast, the marine AOA *N. maritimus* lacks genes coding for citrate lyase and is thus thought to have an incomplete reductive TCA, or only the oxidative TCA cycle [2]. Spang *et al*. (2012) demonstrated the presence of all candidate enzymes for oxidative TCA in the hot spring AOA *Ca*. N. gargensis. Finding of a gene homolog of isocitrate lyase in the *Ca*. N. gargensis genome is evidence of possible usage of the glyoxylate bypass. Replenishment of the TCA intermediates in the *Ca*. N. evergladensis is mediated by either a pyruvate carboxylase or possibly via glyoxylate bypass. Isocitrate lyase as key enzyme of the glyoxylate bypass was identified in the sequenced genome, but malate synthase was not identified.

AOA have a genomic potential to uptake small organic molecules, and the addition of pyruvate stimulates growth of the soil archaeon *N. viennensis* [21]. However, the question of whether AOA are autotrophs or mixotrophs remains unanswered. Green sulfur bacteria can operate the TCA cycle in both directions [33], but compared to autotrophic growth (reductive TCA), green sulfur bacteria prefer mixotrophic growth (oxidative TCA) enhanced with pyruvate. The presence of the gene homologs of the complete oxidative TCA cycle, and encoded amino acid and di- and tricarboxylate transporters in the genome of *Ca*. N. evergladensis suggest the capacity of this AOA to metabolize small organic compounds via this pathway. The presence of 3-hydroxypropionate/4-hydroxybutyrate and reductive TCA cycles in the genome of *Ca*. N. evergladensis may give an advantage to the AOA to survive under different oxygen concentrations. Under the limited oxygen reductive TCA may be used to fix CO_2_ and generate NADH. Reductive TCA is an efficient means to fix CO_2_ (four ATPs per one molecule of pyruvate) but it is an oxygen sensitive pathway. It may operate under restricted oxygen, where NO_2_^−^ produced during aerobic ammonia oxidation may be used as a terminal electron acceptor [34], [35]. Conversely, under high oxygen availability AOA may shift to the less efficient but oxygen insensitive 3-hydroxypropionate pathway (five-nine ATPs per one molecule of pyruvate) [34]. Another evidence that suggests potential of AOA to live in the low-oxygen conditions, where they may operate reductive TCA cycle, is high AOA affinities to oxygen determined in the AOA cultures [9], [16], [36]. If TCA cycle present in *Ca*. N. evergladensis is solely utilized for the biosynthetic purposes, than 3-hydroxypropionate cycle will be the only pathway used for CO_2_ fixation in this archaeon. Herein lies the significance of the results of that Thaumarchaeota can fix CO2 very efficiently under aerobic conditions [29].

#### Gluconeogenesis and glycolysis

*Ca*. N. evergladensis has a complete gluconeogenic pathway (Figure S5 in File S1, Table S1 in File S2). Archaea operate a variety of modified Embden-Meyerhof-Parnas (EMP) pathways, which differ from the classic glycolytic pathway [37]. Unusual enzymes for glycolysis were found in the genome of *Ca*. N. evergladensis such as multiple kinases (NTE_03124, NTE_00636, NTE_01922) from the ribokinase superfamily that have broad substrate specificity (e. g. glucose, fructose and mannose) and can be candidates for hexokinase and phosphofructokinase enzymes for glycolytic pathway in AOA. The isomerization of glucose-6P/fructose-6P in *Nitrososphaera* may be catalyzed by either metal-dependent phosphoglucose isomerase (NTE_01540), which belongs to the cupin superfamily and found in the Euryarchaeota or with bifunctional phosphoglucose/phosphomannose isomerase from the sugar isomerases family (NTE_02296). A homolog of pyruvate dikinase was detected in the genome (NTE_02861). Pyruvate dikinase catalyzes reversible interconversion of PEP and pyruvate in Thermopreotei [33], [37]. Genes encoding glucose-6-phosphate isomerase, sugar kinases and phosphoglycerate kinase were found only in the *Nitrososphaera* species and did not show any close similarity with other Thaumarchaeota.

#### Hexose monophosphate pathway

All enzyme homologs of the non-oxidative phase of the hexose monophosphate pathway were identified in *Ca*. N. evergladensis except 6-phosphogluconate dehydrogenase is missing. This enzyme is one of the key enzymes of the oxidative phase (Figure S6 in File S1, Table S1 in File S2). This enzyme was found in the genome of *Ca*. N. gargensis but not in other Thaumarchaeota. Two other enzymes of the oxidative branch of HMP, F420-dependent oxidoreductase and gluconolactonase, were identified in the genome. F420-dependent oxidoreductase, G6PDH family has been found in archaeal methanogens, *Streptomyces*, and *Mycobacteria* [38], [39]. Similar to other AOA, *Ca*. N. evergladensis does not use the Entner-Doudoroff pathway [3].

### Energy metabolism

**Figure 5:**
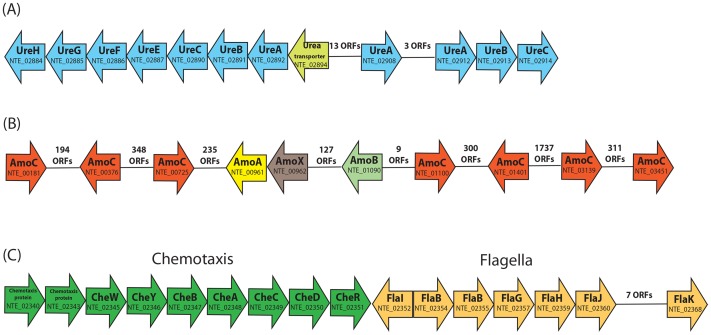
Organization of (A) urease and urea transporter genes; (B) ammonia monooxygenase gene order of *Ca*. N. evergladensis; (C) flagella and chemotaxis genes in the present genome.

An autotrophic lifestyle of AOA, in which NH_3_ and O_2_ are used to generate energy, was demonstrated in multiple studies [1], [30]. Recently it was indicated that a group of polar Thaumarchaeota had the genomic potential to use urea to fuel a key step of nitrification [40], [41]. *Ca*. N. gargensis [20] and *N. viennensis* [21] showed potential to utilize urea as source of NH_3_. Coding sequences for multiple subunits of urease (*ureA*, *ureB*, *ureC*, *ureG*, *ureH*, *ureF*, *ureE*) were found clustered together in the genome with passive and electrochemically-driven urea transporters (Figure 5A). These gene homologs provide evidence that *Ca*. N. evergladensis can use urea as NH_3_ source. Moreover, all subunits of urease have two copies in the genome and were identified only in the *Ca*. Nitrososphaera genomes. Some of AOA from the group I.1a (*Ca*. Nitrosopumilus sp. AR2 and *Cenarchaeum symbiosum*) showed similarity with *Nitrososphaera* ureases [1], [5], [6]. However, the majority of sequenced AOA from the group I.1a (*N. maritimus*, *Ca*. N. limnia, *Ca*. N. salaria, *Ca*. N. koreensis, *Ca*. N. uzonensis) do not have any signatures of urea degradation [1], [4], [16], [19].

Ammonia is oxidized to NO_2_^−^ in a two-step reaction. The first reaction is likely catalyzed by archaeal ammonia momooxygenase (AMO). Several genes (*amoA*, *amoB*, *amoC* and *amoX*-like) are predicted to encode subunits of AMO in the *Ca*. N. evergladensis genome (Figure 5B, Table S1 in File S2). The *amoA* gene is the most conserved of the *amo* genes and shares 99% amino acid identity with *amo* genes of *N. viennensis* and 95% with *Ca*. N. gargensis (Table S1 in File S2). Similar to ammonia-oxidizing bacteria (AOB), archaea of group I.1b encode several *amoC* subunits of AMO [42]. However, majority representatives of group I.1.a have only one copy of *amoC* (Figure S7 in File S1). Little is known regarding the function of *amoC*. Previous studies have revealed that *amoC* may stabilize the AMO under stress conditions such as starvation and heat shock [43]. It is noteworthy that multiple copies of *amoC* appear more often in AOA and AOB associated with soil environments, which harbor more diverse stressors than marine environments and require more adaptations for organisms to survive and successfully compete [42]. For example, ammonia oxidizers from soil have up to seven *amoC* copies (Figure S7 in File S1), while marine AOA and AOB usually encode only one *amoC* [20], [42]. The amino acid alignment identity between seven *amoC* copies ranges between 72% and 97% in the *Ca*. N. evergladensis genome.

Bacterial AMO oxidizes NH_3_ to hydroxylamine (NH_2_OH) and it is further oxidized to NO_2_^−^ by hydroxylamine oxidoreductase (HAO) [44], [45]. However, no homologs of the bacterial HAO are found in AOA genomes [1], [2], [20], [28], [46]. Two hypothetical pathways of NH_3_ oxidation to NO_2_^−^ were proposed [2]. The first suggests nitroxyl (HNO) is produced a reactive intermediate that is further is oxidized to NO_2_^−^ by nitroxyl oxidoreductase. In latter pathway, NH_2_OH is a possible intermediate in the reaction and is oxidized to NO_2_^−^ by periplasmic multicopper oxidases. Recently, Vajrala et al. [47] provided direct evidence that *N. maritimus* oxidizes NH_2_OH to NO_2_^−^. Therefore, the alternative pathway with NH_2_OH as the intermediate is possible. Similar to other AOA, the *Ca*. N. evergladensis genome encodes genes for six periplasmic multicopper oxidase proteins that may be candidates for HAO (Figure S8 in File S1). Moreover, two of these oxidases are dissimilatory copper-containing nitrite reductases (NirK).

AOB channel two electrons from HAO through cytochromes c_554_ to c_m552_ [44]. Similarly to other AOA no homologs for cytochromes c_554_ and c_m552_ were predicted for *Ca*. N. evergladensis. Instead multiple copper-containing plastocyanin-like electron carriers are candidates for transferring electrons to O_2_ (Table S1 in File S2, Figure S8 in File S1). NAD-quinone oxidoreductase (Complex I) catalyzes the transfer of electrons from NADH to ubiquinone. *Ca*. N. evergladensis has 11 genes encoding subunits of NAD-quinone oxidoreductase, but it is missing genes that encode the E, F and G subunits. Further, a proton motive force (PMF) may be generated through complexes III (Rieske Fe-S proteins, plastocyanines), complex IV (proton-pumping oxygen-reducing plastocyanin-copper oxidases), and by complex V (Archaeal/vacuolar-type H^+^-ATPase). Copper-containing nitrite reductases may reduce NO_2_^−^ to nitric oxide (NO). NO was shown to have a stimulating effect on ammonia oxidation in the AOB [48]. Hence, NO may be involved in the regulation of the AMO activity in AOA.

### Triacylglycerols and Polyhydroxyalkanoates as lipid reserve materials

Although many archaea store carbon in the form of polyhydroxyalkanoates (PHAs), some archaea and other organisms preserve carbon in the form of triacylglycerols (TAGs) [49]. *Ca*. N. evergladensis and *Ca*. N. gargensis possess lipases (lysophospholipase, monoglyceride lipase) that may hydrolyze ester bonds in triacylglycerides of long chain fatty acids. Extracellular lipases may also be involved in utilization of monoglycerides from the soil. These lipase homologs are lacking in the group I.1a.

Other lipophilic compounds that likely accumulated in Thaumarchaeota as a reserve material are PHAs. Polyhydroxyalkanoate synthase was found almost in all representatives of Thaumarchaeota [20], [50]. The *Ca*. N. evergladensis genome encodes for class III PHA synthase (*phaC*, *phaE*) (Table S1 in File S2). Gene homolog for subunit PhaE of PHA synthase shares some similarity with *Ca*. N. gargensis, but it is very distantly related to representatives of group I.1a.

#### Isoprenoids as biomarkers for Thaumarchaeota

Archaea use isoprenoids to make phospholipids. The hydrophobic tails of the phospholipids are isoprenoid alcohols ether-linked to glycerophosphate to form monoglycerol-tetraether. Thaumarchaeota have a specific cyclopentane ring-containing dibiphytanyl glycerol tetraether membrane lipid (crenarchaeol) [51], [52]. Damsté et al. [51] hypothesized that formation of cyclohexane ring in crenarchaeol may be an adaptation to cold temperatures in the marine water. However, crenarchaeol was also identified in AOA from thermophilic environments [53]. In addition to crenarchaeol, high concentrations of crenarchaeol regioisomer have been determined in *Ca*. N. gargensis, but this regioisomer is either absent or present in very low amounts in other analyzed AOA from I.1a and ThAOA groups [54]. *Ca*. N. evergladensis has the mevalonate pathway, which operates in archaea and eukaryotes [55]. This pathway is used to synthesize isopentenyl diphosphate (IPP), which is converted to different isoprenoids in the cell (quinones, hydrophobic tails of the phospholipids), using a set of enzymes present in the *Ca*. N. evegladensis genome: farnesyl pyrophosphate synthetase, and octaprenyl pyrophosphate synthetase, undecaprenyl pyrophosphate synthetase (Table S1 in File S2).

### Stress response

The sequenced genome revealed the presence of multiple adaptations to survive osmotic and oxidative stress, high concentrations of heavy metals, and elevated temperatures. Moreover, it contains more diverse mechanisms than AOA from the group I.1a to resist high concentrations of heavy metals.

#### Osmotic stress

One of the strategies to cope with high salinity, and in some cases temperature stress, in archaea is accumulation of compatible solutes, small soluble organic molecules [56]. These solutes can be either transported into the cell or synthesized *de novo*. Several aquaporins that transport water and small uncharged molecules, and belong to the major intrinsic protein family [57], were found in the *Ca*. N. evergladensis genome as well as in other AOA genomes (Table S1 in File S2). Aquaporins identified in *Ca*. N. evergladensis are related to glycerol uptake facilitators. Glycerol is one of the uncharged compatible solutes, which can be used by AOA for osmoadaptation. Mannosyl-3-phosphoglycerate and myo-inositol-1-phosphate synthases were found in the genomes of group I.1b but not in the genomes of group I.1a. These enzymes are involved in the biosynthesis of di-myo-inositol phosphate and mannosylglycerate, two main prokaryotic compatible solutes. These compatible solutes are commonly represented in thermophilic and hyperthermophilic bacteria and archaea [58].

To mitigate oxidative damage, *Ca*. N. evergladensis encodes superoxide dismutase, peroxiredoxins, and ferritin-like proteins. The majority of enzymes involved in oxidative stress response shared similarity with *Ca*. N. gargensis and other AOA. However, some of the peroxiredoxins and ferritin-like proteins (NTE_01148, NTE_01225, NTE_01156) shared sequence similarity to Euryarchaeota and Bacteria but not to Thaumarchaeota (Table S1 in File S2).

#### Resistance to heavy metals

Archaea have been found in extreme environments such as mining sites with high concentration of heavy metals [59], [60]. *Ca*. N. evergladensis developed mechanisms that would help it resist high external concentrations of metals with at least 21 putative heavy metal resistance proteins. Nine of these homologs were encoded only in *Nitrososphaera* genus, and not in other AOA, and six were found only in the *Ca*. N. evergladensis genome (Table S1 in File S2). Both *Ca*. N. evergladensis and *Ca*. N. gargensis are predicted to have broad tolerance to a variety of heavy metals: copper, zinc, cobalt, cadmium, arsenic, and mercury. However, AOA from group I.1a are more limited in their adaptations to high concentrations of heavy metals.

A higher tolerance of AOA than AOB to copper was shown in soil [61]. Ettema *et al*. (2006) [62] suggested a potential copper resistance gene cluster, which consists of a putative methallochaperone and P-type cation transporting ATPase. This mechanism was identified in thermoacidophilic archaeon *Sulfolobus metallicus*, *Sulfolobus solfataricus*, and *Ferroplasma acidarmanus* [63]. A similar gene cluster with encoded P-type ATPases and copper chaperones was found in *Ca*. N. evergladensis (Table S1 in File S2). This mechanism of copper tolerance was also present in *Ca*. N. gargensis genome but not in other sequenced AOA. An alternative putative mechanism of copper detoxification in AOA may involve multicopper oxidases. Multicopper oxidases play an important role in copper resistance in many bacteria [64]. Multiple putative multicopper oxidases are encoded in all known AOA, however, their role in copper tolerance remains unclear (Table S1 in File S2). Periplasmic divalent cation tolerance protein (NTE_02314) is widely represented in AOA, and may also transport copper outside the cell. Copper tolerance may also involve an inorganic polyphosphate transport system [64]. Polyphosphate kinase (PPK), which catalyzes the reversible conversion of the terminal phosphate of ATP into polyphosphates (polyP), and exopolyphosphatase (PPX), is known to hydrolyze polyP. This mechanism was described in other archaea [65]. The enzymes supporting polyP transport encoded in *Ca*. N. evergladensis showed homology to *Methanosarcina*, *Methanoregula*, and *Methanomassiliicoccus* from Euryarchaeota, but this transport is absent from other known Thaumarchaeota.

*Ca*. N. evergladensis has three putative nickel transporter genes, and one of these high affinity permeases (NTE_02909) is specific only for this thaumarchaeon.

Other metal resistance proteins include cobalt-zinc-cadmium resistance proteins (one is unique for *Ca*. N. evergladensis), putative tellurium resistance membrane protein, and arsenic efflux proteins. Notably, an arsenic pump was identified only in the genomes of *Nitrososphaera* species but not in AOA related to *Nitrosopumilus*.

#### Heat shock

Besides the altered composition of lipids in the membranes that are used to survive at elevated temperatures, Thaumarchaeota encode an entire set of proteins to cope with temperature stress [66]. The *Ca*. N. evergladensis genome harbors gene homologs of heat-shock proteins (HSP) such as small HSP, HSP60 (GroEL and Thermosomes), and chaperones such as DnaJ, DnaK and GrpE. Moreover, the copy number of these gene homologs is higher in group I.1b than in group I.1a.

### Nitrogen metabolism

#### Ammonia assimilation

Group I.1b maybe more adapted to high concentrations of NH_3_ than group I.1a. The majority of group I.1a AOA were identified in marine environments (*N. maritimus*, *Ca*. N. koreensis, *Ca*. N. limnia, *Ca*. N. sediminis, *Ca*. N. salaria) where the ammonium concentration was as low as 0.017 mg L^−1^ [23], [67]. Group I.1b is mainly found in the environments at much higher ammonium concentrations (0.1–9 mg L^−1^), such as soil, and representatives of this group are more tolerant of high ammonium levels compared to the majority of AOA isolated from the marine environments or soils with low pH [3], [9], [16], [21], [23], [36].

**Figure 6:**
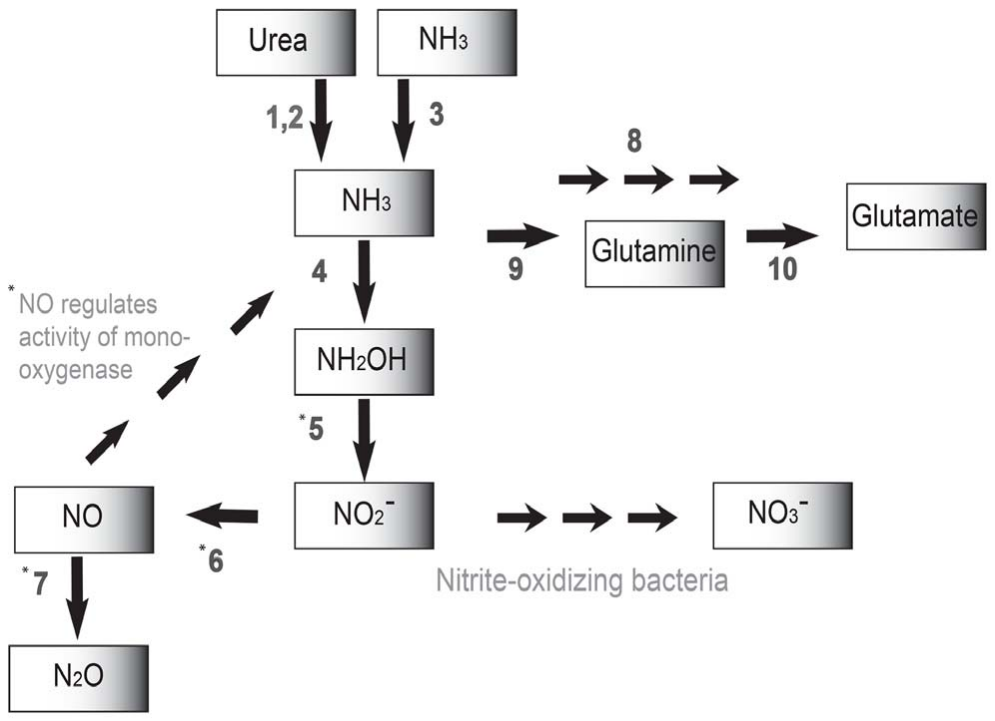
Components of the nitrogen metabolism of *Ca*. N. evergladensis : ammonia oxidation (4, 5), ammonia assimilation (8, 9, 10), nitrite reduction (6), nitrous oxide production (7). Reactions are mediated by the following transporters and enzymes: urea transporters, urease (1, 2), ammonia transporters (3), archaeal ammonia monooxygenase (AMO) (4), candidate enzyme: multicopper oxidase (5), nitrite reductase (NirK) (6), nitric oxide reductase (NorD, NorQ), catalytic subunit (NorB) is missing (7), glutamate dehydrogenase (8), glutamine synthetase (9), glutamate synthase (10). NO may upregulate activity of AMO. * - experimental evidences are needed.

NH_3_ can be used by *Ca*. N. evergladensis not only as energy source, but also as a N source. The full set of enzymes involved in NH_3_ assimilation is present (Figure 6), including glutamate dehydrogenase, glutamine synthetase, and glutamate synthase. Glutamate synthase (NTE_01407) was found only in the *Ca*. N. evergladensis genome, and not other sequenced AOA genomes.

#### Putative pathway for Nitrous oxide production

A cluster of genes that encode a putative multicopper oxidase related to nitrite reductase (*nirK*) and the gene homologs of the nitric oxide reductase subunits (*norD* and *norQ*) are present in the genome of *Ca*. N. evergladensis. However genes coding for the catalytic subunits (*norB* and *norC*) were not identified. Proximity of the *nirK* and *norD,Q* homologs in the genome may suggest that these genes code for the proteins involved in the same metabolic pathway [68]. A similar set of genes was found in both AOA groups (Table S1 in File S2). Nitrite reductase and nitric oxide reductase were shown to be involved in cell tolerance to NO_2_^−^ and NO [69], [70]. Alternatively, as in *Nitrosomonas europaea*, *Ca*. N. evergladensis may use NO_2_^−^ and NO as terminal electron acceptors via a putative denitrification pathway [71]. Several studies have shown that AOA cultures are able to emit N_2_O and several potential pathways were suggested [15]. However, missing intermediates and missing catalytic subunits of enzymes result in an incomplete pathway for N_2_O production, and further experiments are required to determine functional enzymes and intermediates for the N_2_O production pathway [17], [18].

### Transport

In the *Ca*. N. evergladensis genome, 141 transport proteins were identified. (Table S2 in File S2), which is larger than the 89–108 found in group I.1a. Of these, 43 encoded an ATP binding cassette, 17 likely code for pores and channels, and the rest are electrochemical-potential-driven transporters, including the Twin Arginine Translocation system. Twelve of these transporters were not found in other Thaumarchaeota. Di- and tricarboxylate transporters found only in *Ca*. N. evergladensis can be involved in transport of TCA cycle intermediates, such as citrate, malate, and succinate. Thirty-one transporter genes were specific for group I.1b. They include genes coding for mechanosensitive ion channels, urea transporters, a symporter from the major facilitator superfamily (MFS), members of the cation diffusion facilitator family for transport of divalent metals, and members of solute carrier families 5 and 6-like superfamily for co-transport of Na^+^ with sugars, amino acids, inorganic ions, or vitamins. Also unique for group I.1b, were proteins from the sodium bile acid symporter family. Transporters of this family were shown to be involved in sodium-dependent transport of a variety of organic molecules in plants and humans [72], [73].

### Motility, chemotaxis and two-component regulatory systems

At least 69 protein-coding genes related to two-component regulatory systems (TS) were found in *Ca*. N. evergladensis and 70 genes related to TS were found in *Ca*. N. gargensis (Table 1). Notably, group I.1b encodes two and a half times the number of TS genes than group I.1a (Table 1).

**Table 1:** Two component systems (TS) identified in the genomes of AOA representatives from the group I.1a, I.1b and AOB using Conserved Domain Search and TIGRfam database.

Stimulus or related function	TS gene	Group I.1b		Group I.1a			AOB
		*N. everg.*	*N. garg.*	*N. kor.*	*N. lim.*	*N. marit.*	*N. europaea*
Cell-envelope stress	*BaeS*	17	20	8	11	4	6
	*BaeR*	0	0	0	0	0	0
Levels of phosphate	*PhoR*	0(12)*	0(14)	0(6)	0(6)	0(1)	0(5)
	*PhoP*(*PhoB*)	18(3)	21(7)	8(5)	9(9)	11(4)	4(8)
	*PhoU*	18(4)	13(5)	9(3)	10(3)	7(2)	4(1)
Chemotaxis	*CheA*	2	2	0	0	0	3
	*CheB*	2	1	0	1	0	2
	*CheC*	2	1	0	1	0	0
	*CheY*	5	3	0	1	1	3
	*CheR*	0	2	0	3	0	0
	*CheX*	1	0	0	1	0	0
Nitrogen levels	*NtrC*	1(14)	0(24)	0(8)	0(17)	0(6)	1(7)
	*GlnK*	4	4	2	2	2	0
	*NtrB*	1(2)	0(2)	0(1)	0	0(1)	1(3)
Osmotic stress	*EnvZ*	0	0	0	0	0	0
	*OmpR*	1	1	1	0	0	5
Osmotic stress	*MtrB*	0	0	0	0	0	0
	*MtrA*	0(1)	0(1)	0(1)	0(1)	0(1)	0
Trimethylamine N-oxide as an alternate electron acceptor	*TorS*	0(6)	0(7)	0	0(4)	0(4)	0(2)
	*TorR*	0	0	0	0	0	0
Oxidative, weak acid, and osmotic stress	*BarA*	0	0	0	0	0	0
	*SirA*	1	1	1	1	1	2
Copper resistance (CopRS)	*CopS*	0	0(1)	0(1)	0(5)	0(6)	0(5)
	*CopR*	0(2)	0(3)	0(2)	0(2)	0	0(4)
Quorum sensing	*ComP*	0	0	0	0	0	0
	*ComA*	0(2)	0(2)	0	0	0	0
Pyruvate response	*YpdA*	0(4)	0(6)	0(3)	0(3)	0(3)	0(10)
	*YpdB*	0	0	0	0	0	0
	**Total**	69(52)	70(72)	27(30)	26(50)	26(28)	24(45)

* components of TS found by TIGRfam database.

Motility-associated genes involved in archaeal flagella and pili assembly were clustered together with protein-coding genes for chemotaxis (Figure 5C). The operon includes genes encoding flagellins (two copies of *flaB*) followed by *fla*-associated genes (*flaG*, *flaH*, *flaJ*, *flaI*, *flaK*, and *flaD*). Structure and assembly of found flagella are closely related to type IV pili. Proteins involved in motility encoded in the *Ca*. N. evergladensis genome were observed in three other AOA, *Ca*. N. limnia, *Ca*. N. uzonensis and *Ca*. N. gargensis. These flagella-coding genes also share identity with other Thermoprotei (*Desulfurococcus kamchatkensis*, *Ignisphaera aggregans*, *Fervidicoccus fontis*, and *Sulfolobus acidocaldarius*). Adjacent to the *fla*-operon is a set of genes involved in chemotaxis.

Ammonia is the main energy and nitrogen source for AOA. The NtrB/NtrC TS, involved in the response to different NH_3_ levels is present in the *Ca*. N. evergladensis genome (Table 1). NtrB senses the nitrogen levels and, under NH_3_ limitation, activates NtrC by phosphorylation. NtrC activates expression of glutamine synthetase (GlnA) and it allows cells to grow under nitrogen-limited conditions.

The PhoR/PhoP (PhoB) TS is found in the *Ca*. N. evergladensis genome and in other Archaea and Bacteria [74], [75]. It plays an important role for sensing inorganic phosphate levels and under phosphate–limited conditions, PhoR/PhoP activates alkaline phosphatase (Table 1).

The *Ca*. N. evergladensis genome, and other ammonia oxidizers, encode components of TS that provide a respond to different environmental stresses, such as cell-envelope stress (BaeS/BaeR) [76], osmotic pressure (EnvZ/OmpR, MtrAB, BarA/SirA), and copper resistance (CopSR) [77].

*Ca*. N. evergladensis encodes the sensor kinase (YpdA) of TS (YpdA/YpdB) that responds to extracellular pyruvate as a stimulus [78]. This finding supports the hypothesis that pyruvate promotes AOA growth. The response regulator of the TS ComP/ComA, which controls competence in *Bacillus subtilis* via a quorum-sensing mechanism [79], was also found in the group I.1b, but not in the group I.1a.

### Information processing machinery

The information processing machinery of *Ca*. N. evergladensis is similar to other Thaumarchaeota, and it shares more homology with eukaryotes than with bacteria [52], [80], [81] (Table S3 in File S2). The *Ca*. N. evergladensis genome has 61 ribosomal proteins that show a phylum-specific pattern (Table S3 in File S2). One of the specific signatures of the known Thaumarchaeota is that their genomes, including *Ca*. N. evergladensis, are missing gene homologs for r-protein family LXa that is solely present in Archaea. Also, r-proteins L14e and L34e found in other archaeal phyla but not in Thaumarchaeota, are missing from *Ca*. N. evergladensis.

DNA-dependent RNA polymerase II (RNAP) is composed of 12 subunits in *Ca*. N. evergladensis as in *Ca*. N. gargensis [20]. Most of the subunits are homologous to other Archaea [80]. However, in contrast to Euryarchaeota, Crenarchaeota and Nanoarchaeota that have two genes encoding A subunit of RNAP, *Ca*. N. evergladensis and other Thaumarchaeota contain a single *rpoA* gene. This unsplit *rpoA* is common for Eykarya and it was suggested that other archaeal lineages that possess split *rpoA* branched off later in evolution than the Thaumarchaeota [52].

Archaeal RNAP requires two accessory factors: transcription factor B (TFB) (an ortholog of TFIIB), and TATA-box binding protein (TBP) [80]. *Ca*. N. evergladensis has at least nine transcription factors B (TFB), and one TATA-box-binding protein. Other representatives of Thaumarchaeota have a similar number of TFB. For example, *Ca*. N. gargensis and *Ca*. N. koreensis have 11 TFBs, *Ca*. N. limnia encodes at least 9 TFBs, *Ca*. N. sediminis and *N. maritimus* have 10 and 8 TFBs, respectively [2], [20], [28].

Multiprotein bridging factor 1 (MBF1) is a transcriptional cofactor that bridges the TATA box-binding protein (TBP) and regulatory DNA-binding proteins [81]. MBF1 is a conserved protein present in all eukaryotes and archaea, with exception of the *N. maritimus* and *C. symbiosum* [82]. This protein is found within the soil archaea, *Ca*. N. gargensis [20] and *Ca*. N. evergladensis. Apparently, group I.1b branched off evolutionarily earlier than group I.1a, which lost MBF1 over time.

#### DNA replication, repair, cell cycle

The *Ca*. N. evergladensis genome contains three *orc1*/*cdc6* orthologues and one of the *cdc6* orthologs is found only in group I.1b (Table S3 in File S2). *Ca*. N. evergladensis encodes small and large subunits of archaeal DNA polymerase II (pol D) and DNA polymerase type B. Also, it carries genes for the large subunit of replication factor C, both subunits of DNA primase, one copy of archaeal DNA polymerase sliding clamp, DNA ligase, RNase HII, and flap endonuclease. The gene encoding topoisomerase IB, a signature marker for Thaumarchaeota, but not for other archaeal phyla, is present in the *Ca*. N. evergladensis genome.

#### Cell division

Among all archaeal phyla only Thaumarchaeota shares two systems of cell division [52], [82]. One is FtsZ-based, present in bacteria and most archaea, and another is CdvABC-based, which is present in Crenarchaeota, and homologous to the eukaryotic ESCRT system [10]. *Ca*. *N*. evergladensis codes for homologs of CdvA, CdvC (Vps4) and several homologs of CdvB-like proteins (ESCRT-III). Also, the genome has the FtsZ-based division system. *ftsZ* gene homolog encoded in the genome shares 40–63% amino-acid identity with other AOA, and less than 29% with other archaea and bacteria. Another cell division feature shared with eukaryotes and other archaea that is found in *Ca*. N. evergladensis is a homolog of pelota proteins required for meiotic cell division [83]. Pelota homologs are widely represented in archaea [83]. In *Ca*. N. evergladensis pelota homologs have 70% amino acid identity with *Ca*. N. gargensis and 42–50% identity with AOA from the group I.1a (Figure S9 in File S1). In archaea, this protein was suggested to play role in translational elongation, termination, and quality control of mRNA (mRNA surveillance) [84], [85]. Archaeal pelota may be involved in the release of the stalled ribosomes and degradation of damaged mRNA [85].

#### DNA folding and repair

Similar to other Thaumarchaeota, *Ca*. N. evergladensis possesses the genes needed to compress and methylate DNA. The DNA repair system of *Ca*. N. evergladensis includes UvrABC endonuclease, which is common in mesophilic archaea [86]. The soil archaea possess other genes in DNA repair, such as ERCC4-type nuclease and helicase, DNA repair helicase RAD25, nucleotidyltransferase/DNA polymerase involved in DNA repair, and photolyase.

### CRISPR-based system

CRISPRfinder determined only one CRISPR locus 7220 bp in the genome of *Ca*. N. evergladensis. The CRISPR region in *Ca*. N. evergladensis, which is longer than that in *Ca*. N. gargensis and many other AOA, was likely a result of more exposure to viruses in its environment [87]. The CRISPR locus consists of 99 repeat/spacer sequences, which is almost 3 times larger than that of *Ca*. N. gargensis [20]. The CRISPR spacers are 34–38 bp and equally separated with identical 37 bp direct repeats. The two genomes of soil group I.1b had higher CRISPR length and longer repeat length than the marine AOA. The five most common CRISPR-associated proteins (*cas1-4, cas7*) are adjacent to repeat/spacer sequences. Variable sequences or spacers mostly correspond to segments of captured viral sequences [88]. However, only one CRISPR spacer had significant homology to any virus, the *Helicobacter* phage phiHP33. In bacteria CRISPR-Cas system provides resistance to exogenous genetic elements and provides acquired immunity for the cell [88], [89]. Most likely, *Ca*. N. evergladensis utilizes CRISPR in the similar way to maintain genome integrity.

### Phylogeny and adaptations

Do the detected differences between genomes of the AOA from the group I.1a and I.1b may give us clues as to how these organisms have adapted to their environments? Many soil surveys revealed that the analyzed soils across the globe were dominated by AOA from the group I.1b (or *Nitrososphaera* cluster), while marine environments were represented by AOA from the group I.1a (or *Nitrosopumilus* cluster) [7], [23]. Auguet et al. [23] studied global ecological patterns of Archaea and found that habitat classification was a strong structuring factor of the archaeal communities. Cells of isolated AOA from group I.1a are typically straight rods [3], [19], [90], [91], whereas cultured I.1b archaea are spherically shaped [16], [20], [21]. Another important physiological difference between two lineages is preference of ammonia concentrations discussed above. Analysis of genomic features of *Nitrososphaera* genus may point to other physiological signatures of this group. For example, sequenced AOA representatives from the group I.1b have a larger genome size and higher G+C content than the archaea from the group I.1a.

More than 3,000 CDS are exclusively present in the genomes of Thaumarchaeota from the group I.1b but are absent in the genomes from the group I.1a AOA (Figure 3B). Coding sequences unique to the I.1b archaea included DNA repair proteins, transporters, two-component systems, and information processing machinery (Table S4 in File S2). Enzymes involved in DNA repair unique to *Ca*. Nitrososphaera included DNA repair photolyase, predicted DNA alkylation repair enzyme, an uncharacterized protein predicted to be involved in DNA repair, and replicative and repair DNA polymerase IV (family X). Also, group I.1b uniquely possessed some proteins involved in information processing machinery, such as DNA topoisomerase IA, ribosomal protein L6p, and transcriptional regulators.

The central metabolism of the AOA from the group I.1b is functionally more diverse than that of I.1a group AOA. AOA from the group I.1b have complete TCA cycle and HMP pathways. Also, unlike the I.1a group, the I.1b group seems to be capable of utilizing complex carbohydrates such as glycogen, chitin, and triacylglycerides, as suggested by the presence of genes coding for the glycogen debranching enzyme (NTE_01977), multiple chitin deacetylases, gene homologs for chitinases (NTE_00025, NTE_01408), and monoglyceride lipases. This appears appropriate given the oligotrophic nature of the environments where representatives of I.1a were isolated.

The I.1b group AOA also possess more transporters than I.1a group AOA, such as an ATPase P-type transporter, a urea transporter, a putative hydroxymethylpyrimidine transporter CytX, Di- and tricarboxylate transporters, and transporters from the solute carrier families 5 and 6-like superfamily. These transporters suggest that the compounds transported by these systems are available in the soil environment, but absent or rare in the marine environment.

Several transposases from family IS605 were encoded in the AOA genomes from the group I.1b, but not in the group I.1a. Transposable elements are widely distributed in archaeal genomes, and play an important role in the genome plasticity and response to environmental stimuli [92].

**Figure 7:**
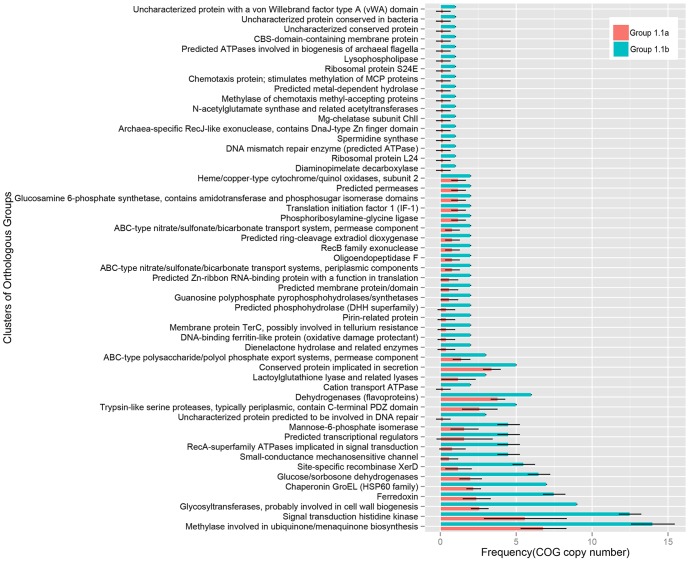
Distribution of Clusters of Orthologous Groups (COGs) in the AOA genomes from soil group I.1b and I.1a. COGs represented with higher copy number in the group I.1b than in the group I.1a were selected by Ttest (*P*_value_<0.05). Error bars display standard deviation.

The genomes from the group I.1b also have a higher number of gene duplications compared to the genomes from the group I.1a (Figure 7). Most of the duplicated genes in the group I.1b are involved in adjusting to different environmental conditions, responses to environmental stresses, or efficient nutrient utilization.

### Provisional classification and conclusion

In this study we sequenced and analyzed genome of the mesophilic AOA from the group I.1b enriched from the soil. We propose the following *Candidatus* status for this microorganism:

“Nitrososphaera evergladensis” sp. nov.

#### Ethymology

Nitrosus (Latin masculine adjective), nitrous, produces nitrite; sphaera (Latin feminine. n.), spherically shaped; evergladensis (Latin neutrum genitive), isolated from the Everglades.

#### Locality

Histosol from the Everglades agricultural area.

#### Diagnosis

Ammonia-oxidizing archaea phylogenetically related to the Thaumarchaeota group I.1b (*Nitrososphaera* cluster) [93]; not isolated; enriched from the agricultural soil.

Analysis of the *Ca*. N. evergladensis genome revealed many similarities of basic metabolism with the rest of AOA, including genes coding for ammonia transporters and genes for AMO subunits, genes for CO_2_ fixation via modified hydroxypropionate cycle, as well as the HMP, TCA and gluconeogenic pathways. This organism belongs to the group I.1b of the Thaumarchaeota, and shares most of its coding sequences with the closest sequenced relative, *Ca*. N. gargensis, isolated from hot springs. Despite the fact that *Ca*. N. evergladensis is phylogenetically closely related to *Ca*. N. gargensis, they have only 40% of whole genome homology revealing significant differences in the metabolic potential of these organisms. The majority of CDS present in *Ca*. N. evergladensis, but absent in *Ca*. N. gargensis, are hypothetical proteins (Table S5 in File S2). *Ca*. N. evergladensis is also distinct from its closest relative, *Ca*. N. gargensis in that it has a much larger CRISPR region, CRISPR-associated genes, transporters for inorganic and small organic molecules, electron carriers, steroid isomerases, chitin deacetylases, and transcriptional regulators that are completely absent in the *Ca*. N. gargensis genome.

When we compared the genetic potential of the archaeal ammonia oxidizers from group I.1b and group I.1a, the AOA from the group I.1b demonstrated a higher potential to adapt to changes in the environment, and to utilize a broad array of carbon sources compared to the AOA representatives from the group I.1a.

About half of all identified proteins were not assigned to functions and may encode completely novel pathways. Further experiments must be conducted to link novel genes to their specific functions, and determine their ecological role.

## Materials and Methods

These soil samples were not collected at a national park or private land. The land is owned by the University of Florida and is within the Everglades Agricultural Area, not the Everglades National Park. No permits were required to collect the soil samples uses in this work. The field studies did not involved endangered or protected species. The GPS coordinates of the research site are: 26.667863, −80.633039.

### Enrichment culture

Soil samples for the enrichment were collected from agricultural plots in the Everglades Agricultural Area planted with sugarcane. The soil from this location in the Everglades Agricultural Area is classified as a histosol with pH ∼8, moisture ∼123%, organic matter ∼70%, nitrate concentration ∼54 mg per kg soil, and ammonium concentration ∼9 mg per kg soil. To enrich for *Ca*. N. evergladensis, 10 g of soil were resuspended in 0.5 L of the medium for culturing of ammonia-oxidizing archaea (AOA) [21]. The medium contained 0.5 mM NH_4_Cl, and 2 ml NaHCO_3_ (1M). The headspace above the non-shaking culture was air. One fifth of the enrichment culture was transferred to fresh medium every four weeks for over one year. To further enrich the medium, several antibiotics, including gentamicin (50 µg/ml), tetracycline (5 µg/ml), and erythromycin (10 µg/ml), were applied in order to suppress growth of co-cultured bacteria. However, the addition of antibiotics also affected archaea and did not produce a pure AOA culture. The concentrations of NH_3_ and NO_2_^−^ were determined by Griess Reagent Kit for Nitrite Determination (G-7921) (Molecular Probes, Eugene, OR, USA), and by the Ammonia Assay Kit (Sigma, St. Louis, MO, USA).

### Extraction of DNA

Cells were collected by filtering 1L of culture onto 0.1 µm polycarbonate membrane (Millipore; Billerica, MA, USA). DNA was isolated from the membrane using the PowerSoil DNA Isolation Kit (MO BIO; Carlsbad, CA, USA). Extractions were performed according to the manufacturer's protocol. All genomic DNA concentration and purity were determined by NanoDrop spectrophotometry (Thermo Scientific; Wilmington, DE, USA) and by Qubit 1.27 Fluorometer (Invitrogen; Grand Island, NY, USA).

### Quantification of archaeal 16S rRNA and *amoA* genes

Bacterial and archaeal 16S rRNA genes were amplified using universal prokaryotic primers 515F (5^'^-GTGCCAGCAGCCGCGGTAA-3^'^ ) and 806R (5^'^-GGACTACVSGGGTATCTAAT-3^'^) [94], cloned into pCR4-TOPO vector and sequenced with M13f and M13r vector primers using Sanger sequencing standard protocol. The archaeal *amoA* copy number in the culture was measured by quantitative PCR (qPCR). Primer sets Arch-amoAf and Arch-amoAr were used [95] (File S1 and S2). Bacterial *amoA* detection was carried out using primer set AmoA1f and AmoA2r [96].

### DNA sequencing

Enrichment culture was sequenced using an Ion Torrent Personal Genome Machine (PGM) (Life Technologies; Grand Island, NY, USA), and the Pacific Biosciences platform (Pacific Biosciences; Menlo Park, CA, USA), according to the manufacture's protocols. Ion Torrent sequencing resulted in 2,389,864 reads with average read length 241 bp (∼127X coverage). PacBio platform produced 197,138 reads with an average length 4,117 bp (∼179X coverage) (Table B in File S1).

### Genome assembly and annotation

Sequenced Ion Torrent reads were imported into CLC Genomics Workbench v.4.0.3 (CLC bio; Aarhus, Denmark), and quality trimmed using a minimum phred score of 20 (with a limit of 5% of low quality bases per read) and a minimum read length of 80 bp. PacBio reads were processed with BLASR mapper (http://www.pacbiodevnet.com/SMRT-Analysis/Algorithms/BLASR), and filtered by size. Ion Torrent reads were independently assembled with two *de novo* assemblers IDBA-UD [97] and Mira 3.9 [98], which resulted in 212 contigs with a length up to 41,248 bp, and 24 contigs with a maximum length 418,142 bp, respectively (Table C in File S1). PacBio reads were assembled using Mira 3.9 [98] and Celera from SMRT portal (http://www.pacbiodevnet.com/SMRT-Analysis/Software/SMRT-Pipe) assemblers, which yielded 21 contigs with maximum length 15,072 bp, and one contig 2,954,373 bp, respectively. In addition, all assembly results were compared and verified for errors using Vista [99] and Mauve [100] tools. Custom primers were designed to experimentally confirm complete genome assembly. Random regions with high fluctuations of G+C content, non-coding regions between operons, and regions with contig overlaps were verified by PCR amplifications. The assembled genome was annotated by the Rapid Annotations using Subsystems Technology (RAST) [101] and Expert Review version of the Integrated Microbial Genomes system (IMG ER) [102]. Limited inspection and clean up of coding sequences was done by comparison with the publicly available databases GenBank [103], TIGRfam [104], the database of Clusters of Orthologous Groups of proteins (COGs) [105], and Conserved Domain Database (CDD) [106]. CRISPRFinder was used to identify CRISPR loci [107]. The Conserved Domain Search tool was used for the annotation of two-component systems. The results from two different databases: TIGRfam and CDD were compared and merged together. Detailed information on the annotated genes can be found in Tables S1–S3 in File S1.

### Phylogenetic analyses

Amino acid sequences of *amo* genes and nucleotide sequences of 16S rRNA were aligned using MUSCLE 3.8.31 [108]. GBLOCKS were used to select conserved sites and remove poorly aligned regions [109]. Likelihood trees were built using PhyML [110]. The optimized parameters for 16S rRNA and for AMO protein sequences are described in File S1 and S2.

### Genome synteny, average nucleotide identity and whole-genome homology

The genome synteny plots were generated from pairwise alignments between the present genome and *Ca*. N. gargensis, and *N. maritimus* genomes obtained from GenBank database. The alignments were based on the six-frame amino acid translations of the compared genomes using Promer tool from MUMmer 3.0 system [111]. The JSpecies software was used to calculate average nucleotide identity between genomes based on the MUMmer ultra-rapid aligning tool [112]. Whole-genome homology was determined from alignment of whole genomes by VISTA servers [99].

### Identification of unique coding sequences for group I.1a and I.1b (Venn Diagrams)

Coding sequences (CDS) of six sequenced AOA genomes were downloaded from GenBank. All CDS from the group I.1a were merged together and CDS from the group I.1b were also merged together. Redundant CDS were removed by clustering sequences from the group I.1a and I.1b at 50% identity using UCLUST v1.2.22q and choosing only unique sequence from each cluster [113]. A protein BLAST of sequences from group I.1b versus group I.1a was performed to determine shared, and unique CDS for both groups at ≥35% identity.

### Sequence deposition

The genome sequence of “*Ca*. Nitrososphaera evergladensis” has been submitted to GenBank under Accession Number CP007174.

## Supporting Information

File S1 Combined file containing Figures S1–S9 and Tables A–C. **Figure S1. Circular representation of the** ***Ca*** **. Nitrososphaera evergladensis genome** (A). From outside to the center: Genes on forward strand (color by COG categories); Genes on reverse strand (color by COG categories); RNA genes (tRNAs green, rRNAs red, other RNAs black); GC content; GC skew. Alignment between Mira contigs generated from Ion Torrent reads and Celera contig generated from PacBio reads (B). Vertical colored lines indicate a high alignment score and white lines indicate a low score. **Figure S2. A phylogenetic tree of ammonia-oxidizing archaea** ***amoA*** **,** ***amoB*** **,** ***amoC*** **, and** ***amoX*** **subunits of ammonia monooxygenase.** Amino-acid sequences of amo subunits of AOA were randomly selected from the National Center for Biotechnology Information databases. The multiple sequence alignment of the amino-acid sequences was used for building maximum-likelihood trees. The branching patterns are denoted by their respective bootstrap values (100 iterations). Topology is colored by the metabolic group (blue represents marine group I.1a, green represents group I.1b, red is ThAOA). **Figure S3. 3-Hydroxypropionate cycle.** Identified enzymes in *Ca*. N. evergladensis genome are in green color; missing enzymes are in red color. **Figure S4. TCA cycle.** Identified enzymes in *Ca*. N. evergladensis genome are in green color; missing enzymes are in red color. **Figure S5. Gluconeogenesis/Glycolysis.** Identified enzymes in *Ca*. N. evergladensis genome are in green color; candidates for enzymes are in red color. **Figure S6. Hexose monophosphate pathway (HMP).** Identified enzymes in *Ca*. N. evergladensis genome are in green color; missing enzymes are in red color. **Figure S7. Clustering of the** ***amo*** **genes coding for subunits of Ammonia monooxygenase (AmoA, AmoB, AmoC, AmoX) in the genomes of ammonia-oxidizing archaea (AOA) and ammonia-oxidizing bacteria (AOB).** **Figure S8. Electron transport chain of** ***Ca*** **. N. evergladensis.** AMO – ammonia monooxygenase; CuHAO – hydroxylamine oxidoreductase; NIR- nitrite reductase; NOR – nitric oxide reductase; PC- small blue copper-containing plastocyanin-like electron carriers; Q and QH_2_ – oxidized and reduced quinone pools. Complex I - Quinone reductase; Complex III – Riske Fe-S proteins, cytochromes; Complex IV – Heme/copper-type cytochrome/quinol oxidases. * - Suggested candidate enzymes: CuHAO (multicopper oxidase), NOR (catalytic subunits NorD, Q are not found). **Figure S9. A phylogenetic tree of archaeal pelota gene homologs.** Amino-acid sequences of pelota were randomly selected from the National Center for Biotechnology Information databases. The multiple sequence alignment of the amino-acid sequences was used for building maximum-likelihood trees. **Table A**. Comparison of *Ca*. N. evergladensis with other AOA genomes that are available in the public databases. CDS were compared at amino-acid identity ≥35%. **Table B**. Sequencing reports from the Ion Torrent platform and Pacific Biosciences platform. **Table C**. Comparative results of different assembly methods and sequencing technologies.
(PDF)

File S2 Combined file containing Tables S1–S5. **Table S1**. Protein coding sequences of central carbon, nitrogen, lipid metabolism and genes involved in the stress response of the archaeon. **Table S2**. Transporters encoded in *Ca*. N. evergladensis genome. **Table S3**. Information processing machinery of *Ca*. N. evergladensis. **Table S4**. Protein coding sequences (COGs and TIGRfams) present only in the AOA group I.1b. **Table S5**. Coding sequences present only in *Ca*. N. evergladensis genome but missing from the genome of *Ca*. N. gargensis.
(XLSX)
