# Supplementary material for: Genome Sequence of Candidatus Nitrososphaera evergladensis from Group I.1b Enriched from Everglades Soil Reveals Novel Genomic Features of the Ammonia-Oxidizing Archaea
Source: PLoS One. 2014 Jul 7;9(7):e101648. doi: 10.1371/journal.pone.0101648 (PMC4084955; doi:10.1371/journal.pone.0101648)
Supplement: File S1 — Combined file containing Figures S1–S9 and Tables A–C. Figure S1. Circular representation of the Ca . Nitrososphaera evergladensis genome (A). From outside to the center: Genes on forward strand (color by COG categories); Genes on reverse strand (color by COG categories); RNA genes (tRNAs green, rRNAs red, other RNAs black); GC content; GC skew. Alignment between Mira contigs generated from Ion Torrent reads and Celera contig generated from PacBio reads (B). Vertical colored lines indicate a high alignment score and white lines indicate a low score. Figure S2. A phylogenetic tree of ammonia-oxidizing archaea amoA , amoB , amoC , and amoX subunits of ammonia monooxygenase. Amino-acid sequences of amo subunits of AOA were randomly selected from the National Center for Biotechnology Information databases. The multiple sequence alignment of the amino-acid sequences was used for building maximum-likelihood trees. The branching patterns are denoted by their respective bootstrap values (100 iterations). Topology is colored by the metabolic group (blue represents marine group I.1a, green represents group I.1b, red is ThAOA). Figure S3. 3-Hydroxypropionate cycle. Identified enzymes in Ca. N. evergladensis genome are in green color; missing enzymes are in red color. Figure S4. TCA cycle. Identified enzymes in Ca. N. evergladensis genome are in green color; missing enzymes are in red color. Figure S5. Gluconeogenesis/Glycolysis. Identified enzymes in Ca. N. evergladensis genome are in green color; candidates for enzymes are in red color. Figure S6. Hexose monophosphate pathway (HMP). Identified enzymes in Ca. N. evergladensis genome are in green color; missing enzymes are in red color. Figure S7. Clustering of the amo genes coding for subunits of Ammonia monooxygenase (AmoA, AmoB, AmoC, AmoX) in the genomes of ammonia-oxidizing archaea (AOA) and ammonia-oxidizing bacteria (AOB). Figure S8. Electron transport chain of Ca . N. evergladensis. AMO – ammonia monooxygenase; CuHAO – [file pone.0101648.s001.pdf]

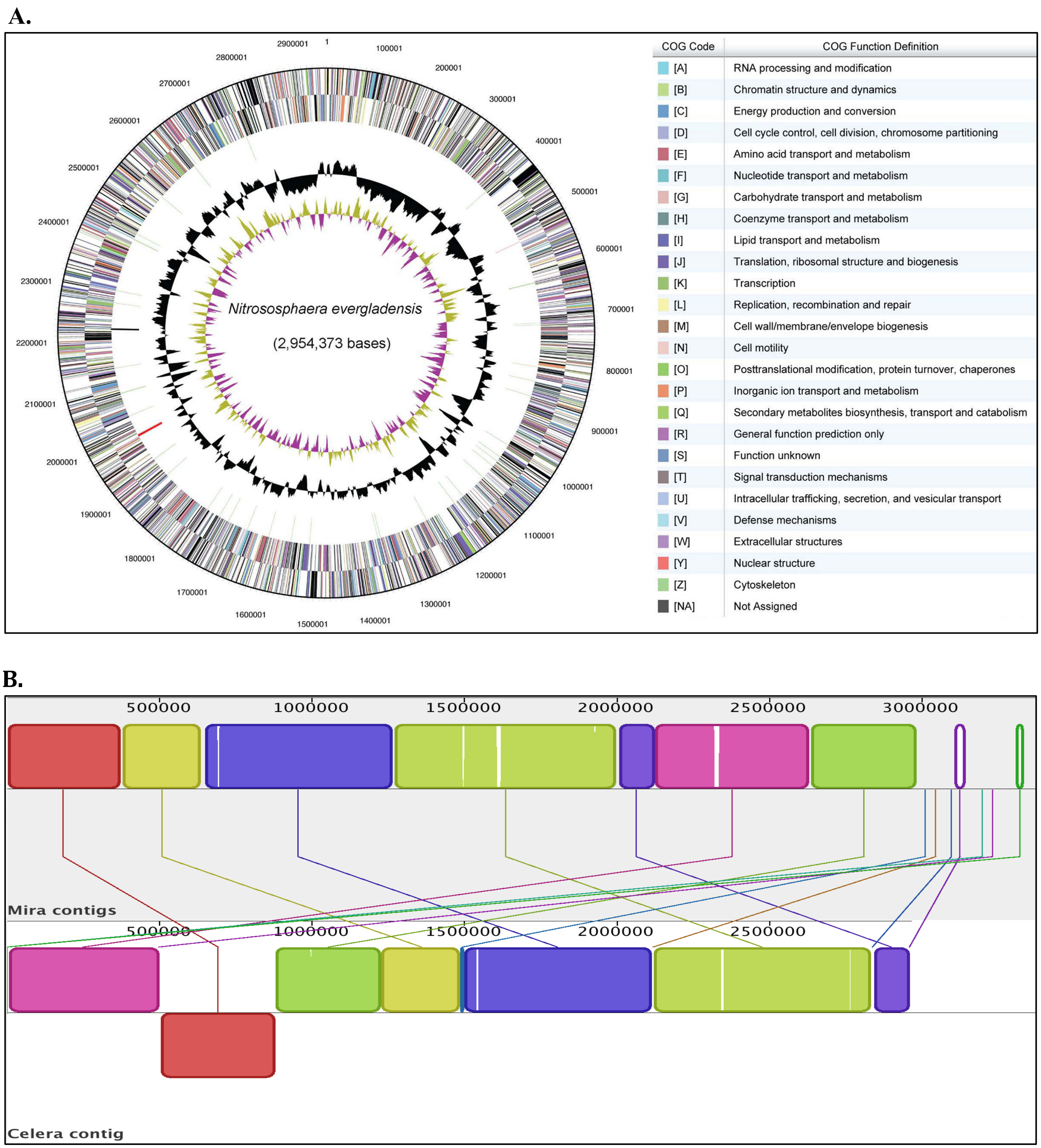

Figure S1. Circular representation of the *Ca*. Nitrososphaera evergladensis genome (A). From outside to the center: Genes on forward strand (color by COG categories); Genes on reverse strand (color by COG categories); RNA genes (tRNAs green, rRNAs red, other RNAs black); GC content; GC skew. Alignment between Mira contigs generated from Ion Torrent reads and Celera contig generated from PacBio reads (B). Vertical colored lines indicate a high alignment score and white lines indicate a low score.

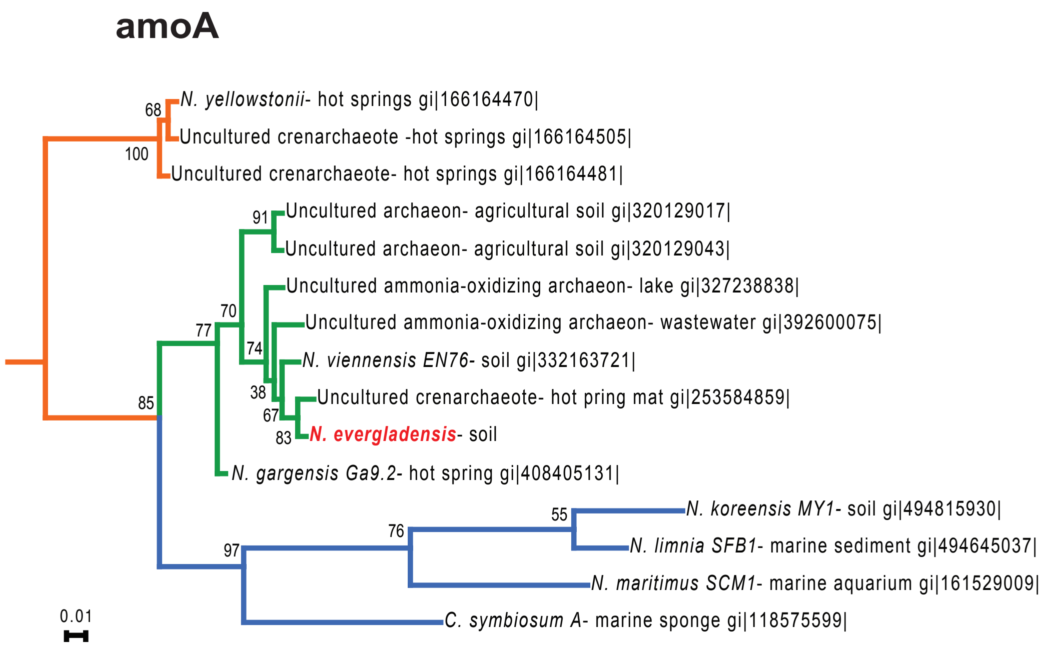
amoA
N. yellowstonii- hot springs gi|166164470|
Uncultured crenarchaeote -hot springs gi|166164505|
Uncultured crenarchaeote- hot springs gi|166164481|
Uncultured archaeon- agricultural soil gi|320129017|
Uncultured archaeon- agricultural soil gi|320129043|
Uncultured ammonia-oxidizing archaeon- lake gi|327238838|
Uncultured ammonia-oxidizing archaeon- wastewater gi|392600075|
N. viennensis EN76- soil gi|332163721|
Uncultured crenarchaeote- hot pring mat gi|253584859|
N. evergladensis- soil
N. gargensis Ga9.2- hot spring gi|408405131|
N. koreensis MY1- soil gi|494815930|
N. limnia SFB1- marine sediment gi|494645037|
N. maritimus SCM1- marine aquarium gi|161529009|
C. symbiosum A- marine sponge gi|118575599|
68
100
91
70
77
74
38
67
83
85
55
76
97
0.01

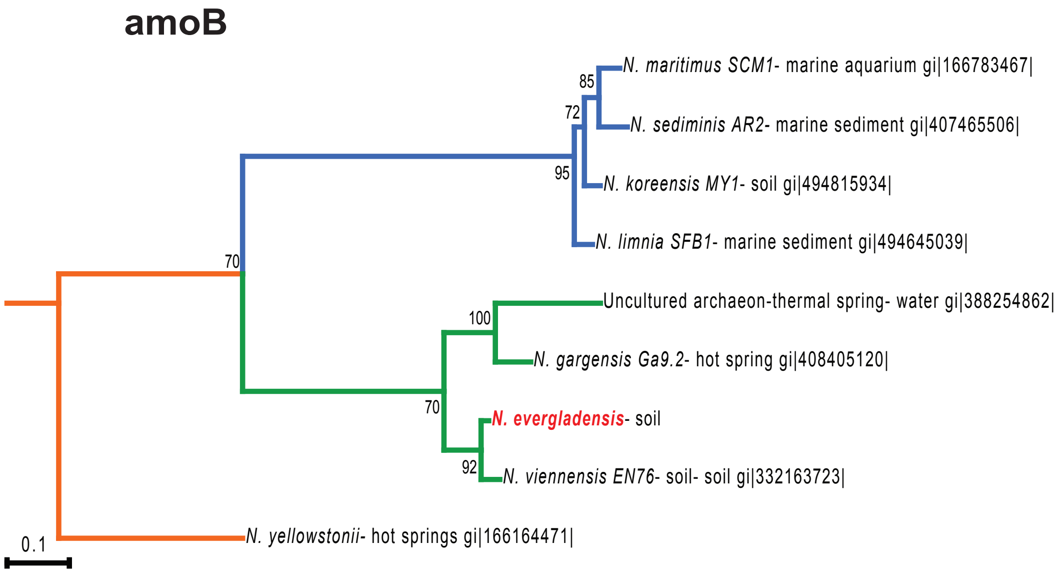
amoB
N. maritimus SCM1- marine aquarium gi|166783467|
N. sediminis AR2- marine sediment gi|407465506|
N. koreensis MY1- soil gi|494815934|
N. limnia SFB1- marine sediment gi|494645039|
Uncultured archaeon-thermal spring- water gi|388254862|
N. gargensis Ga9.2- hot spring gi|408405120|
N. evergladensis- soil
N. viennensis EN76- soil- soil gi|332163723|
N. yellowstonii- hot springs gi|166164471|
85
72
95
70
100
70
92
0.1

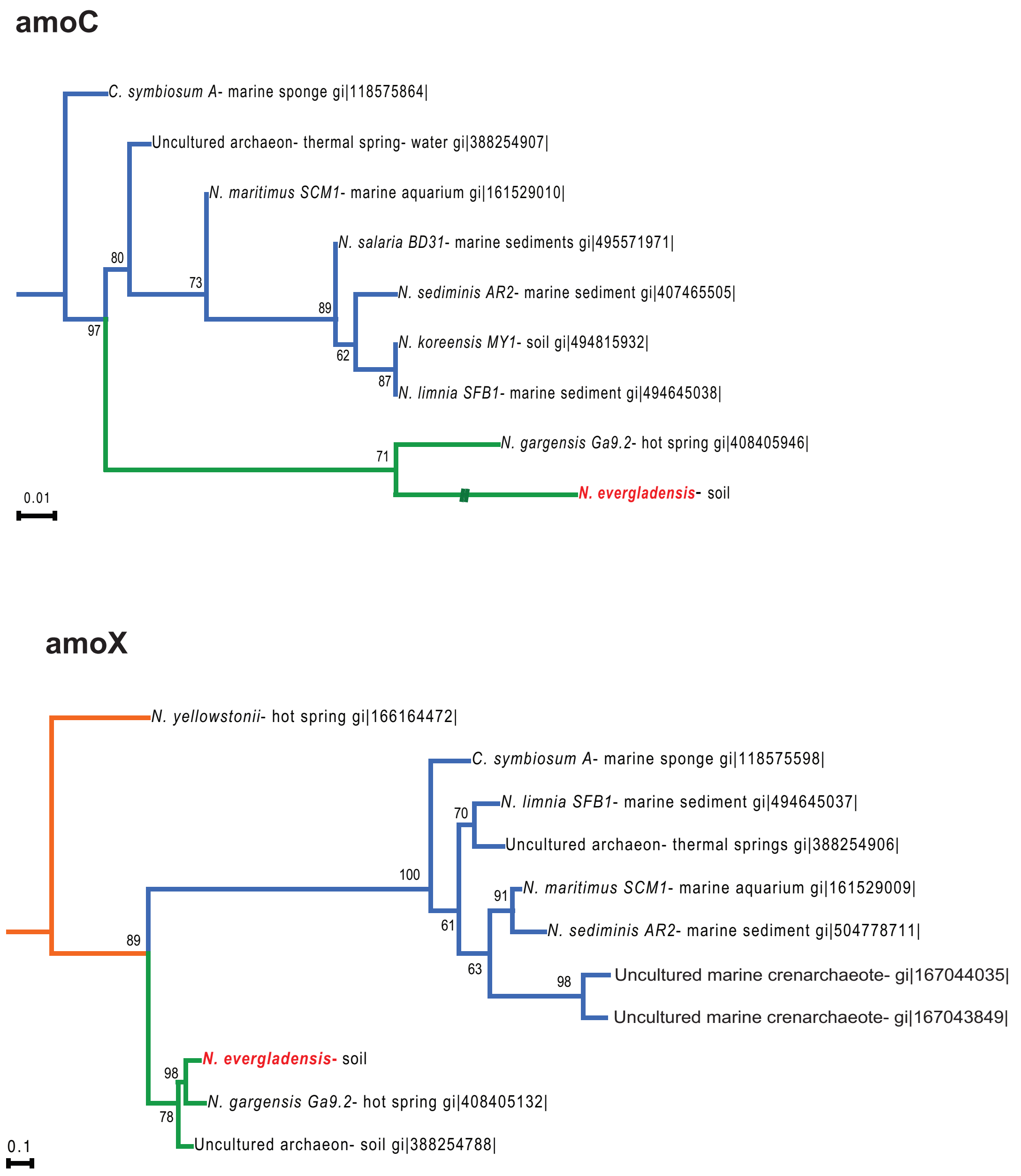

Figure S2. A phylogenetic tree of ammonia-oxidizing archaea amoA, amoB, amoC, and amoX subunits of ammonia monooxygenase. Amino-acid sequences of amo subunits of AOA were randomly selected from the National Center for Biotechnology Information databases. The multiple sequence alignment of the amino-acid sequences was used for building maximum-likelihood trees. The branching patterns are denoted by their respective bootstrap values (100 iterations). Topology is colored by the metabolic group (blue represents marine group 1.1a, green represents group 1.1b, red is ThAOA).

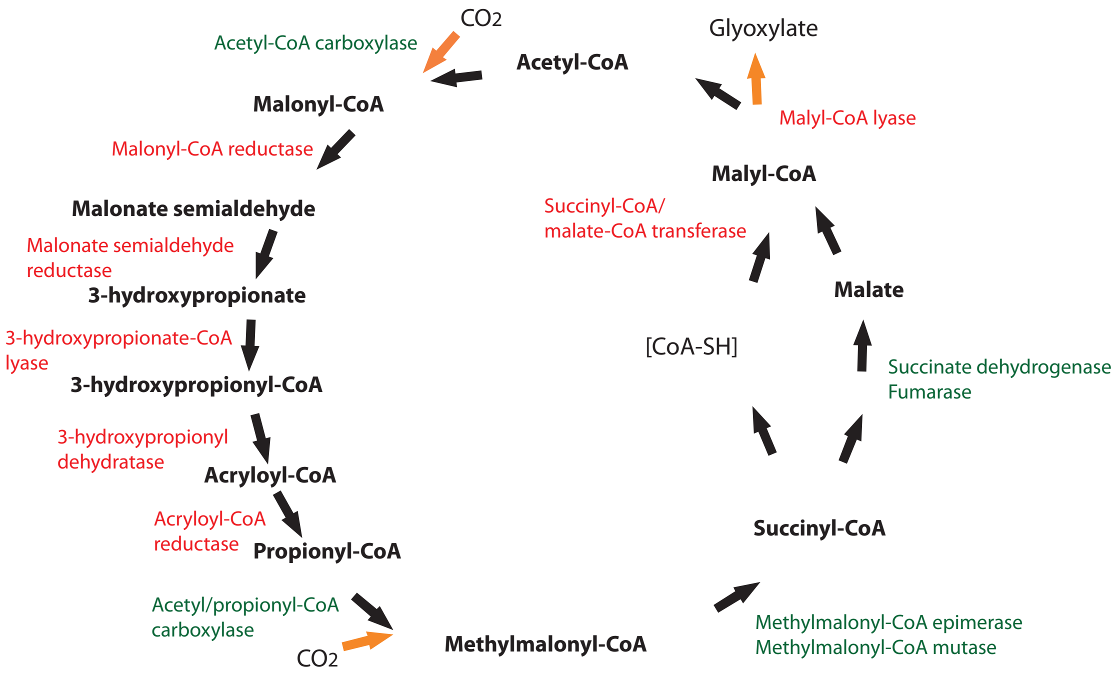

Figure S3. 3-Hydroxypropionate cycle. Identified enzymes in *Ca*. N. evergladensis genome are in green color; missing enzymes are in red color.

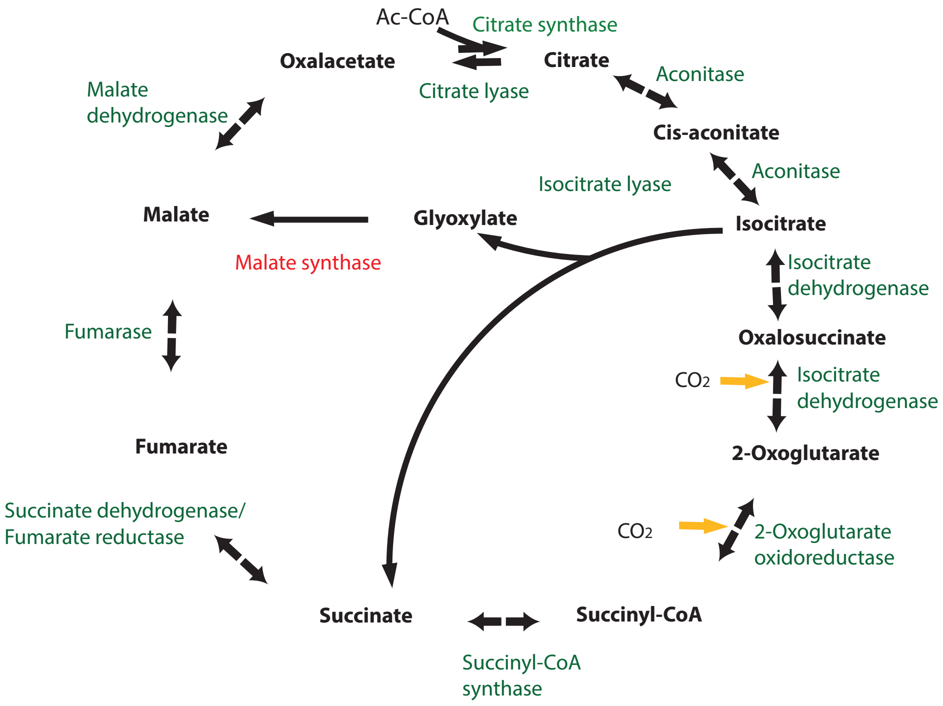

Figure S4. TCA cycle. Identified enzymes in *Ca*. N. evergladensis genome are in green color; missing enzymes are in red color.

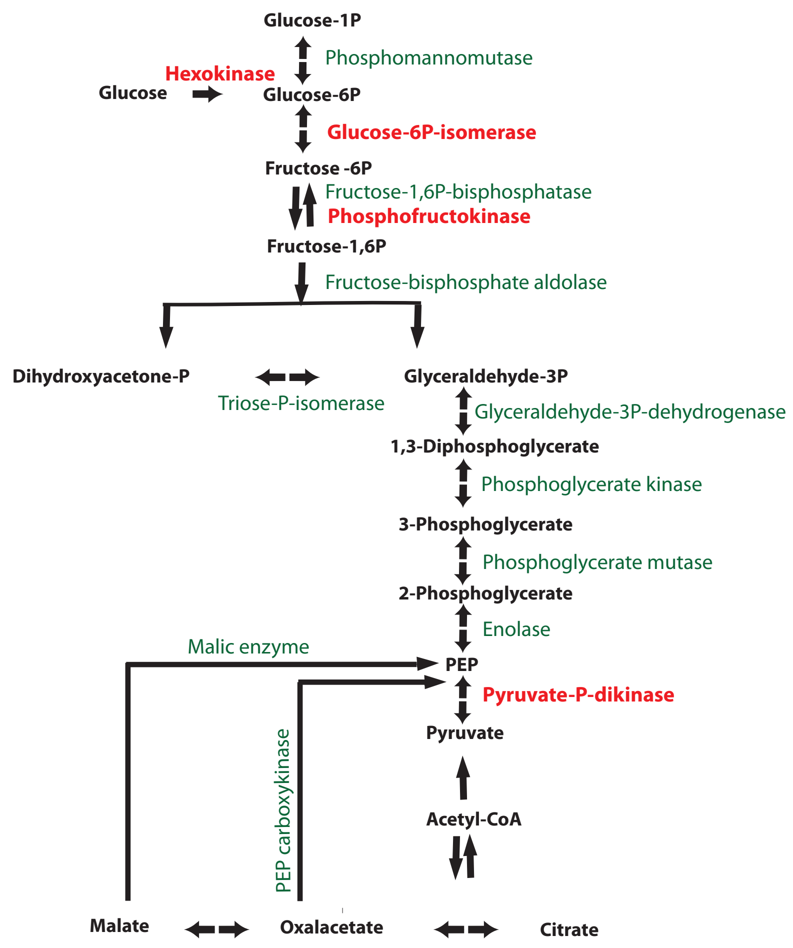

Figure S5. Gluconeogenesis/Glycolysis. Identified enzymes in *Ca*. N. evergladensis genome are in green color; candidates for enzymes are in red color.

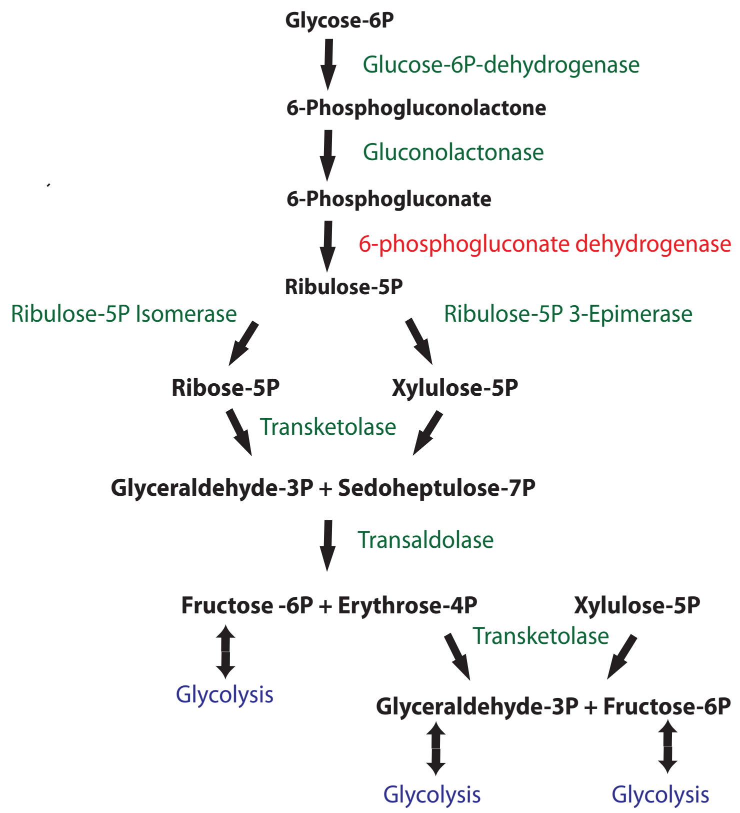

Figure S6. Hexose monophosphate pathway (HMP). Identified enzymes in *Ca*. N. evergladensis genome are in green color; missing enzymes are in red color.

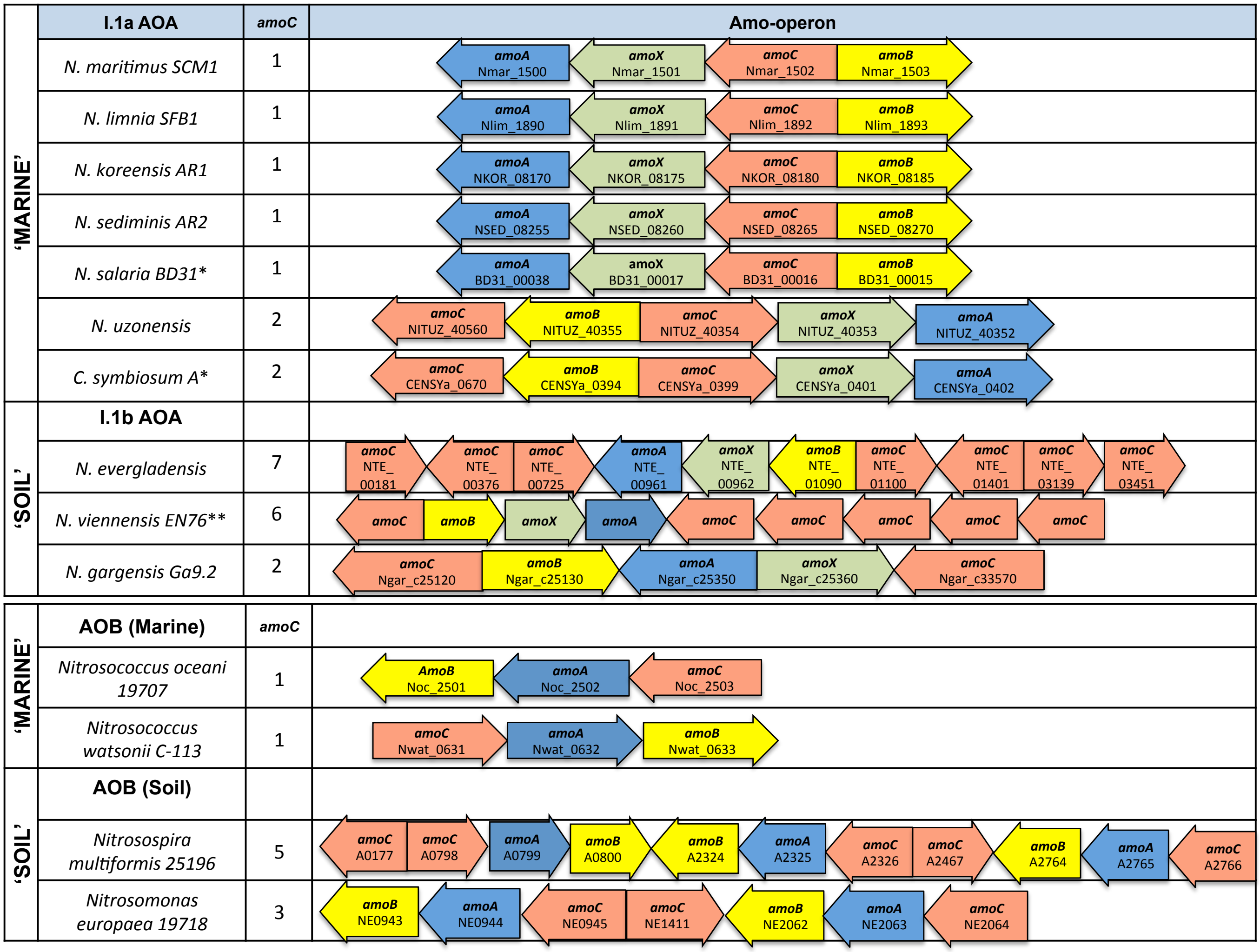

* has not been shown to oxidize ammonia
** Spang *et al.*, 2010

Figure S7. Clustering of the *amo* genes coding for subunits of ammonia monooxygenase (AmoA, AmoB, AmoC, AmoX) in the genomes of ammonia-oxidizing archaea (AOA) and ammonia-oxidizing bacteria (AOB).

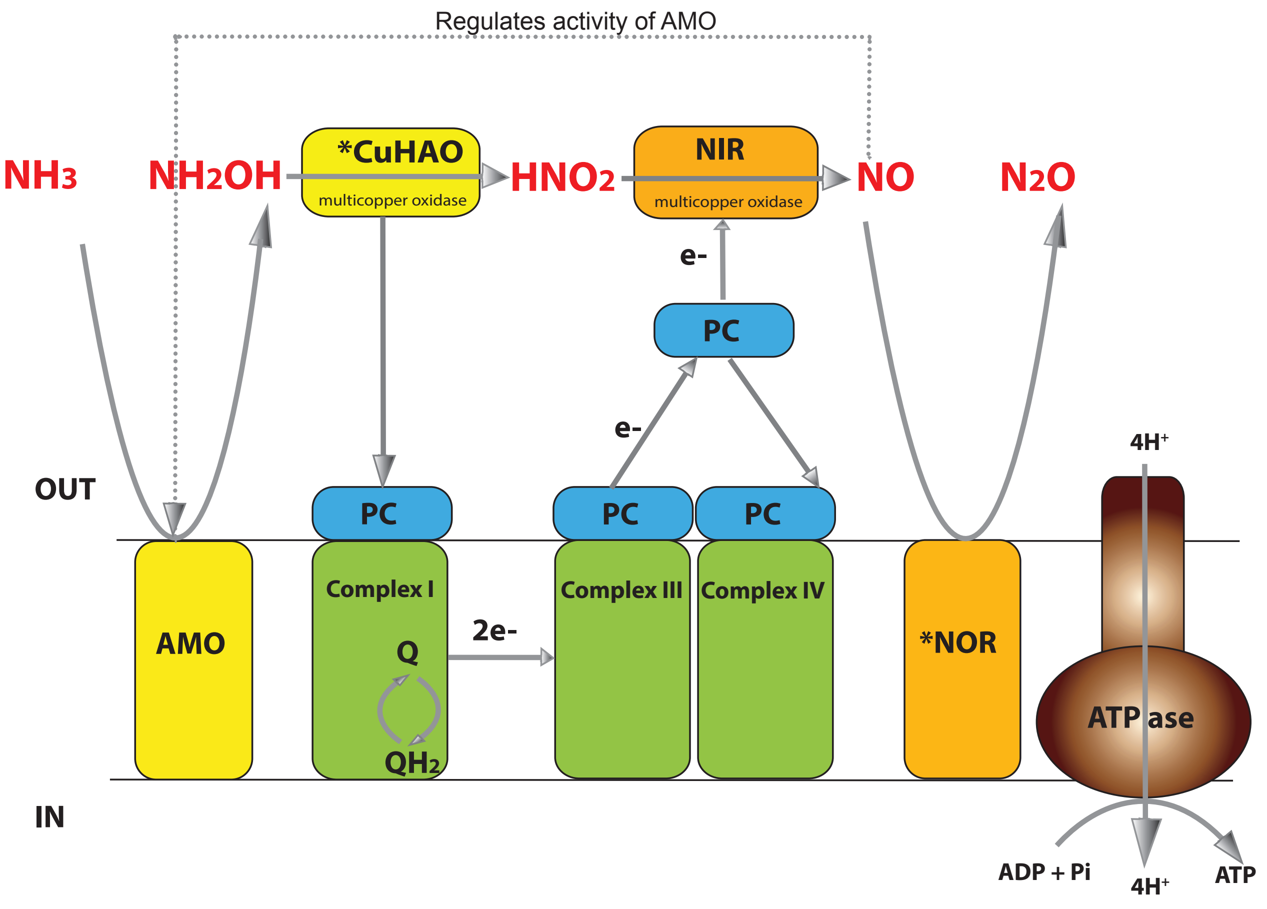

Figure S8. Electron transport chain of *Ca*. N. evergladensis. AMO – ammonia monooxygenase; CuHAO – hydroxylamine oxidoreductase; NIR- nitrite reductase; NOR – nitric oxide reductase; PC- small blue copper-containing plastocyanin-like electron carriers; Q and QH_2 – oxidized and reduced quinone pools. Complex I - Quinone reductase; Complex III – Riske Fe-S proteins, cytochromes; Complex IV – Heme/copper-type cytochrome/quinol oxidases. * - Suggested candidate enzymes: CuHAO (multicopper oxidase), NOR (catalytic subunits NorD, Q are not found).

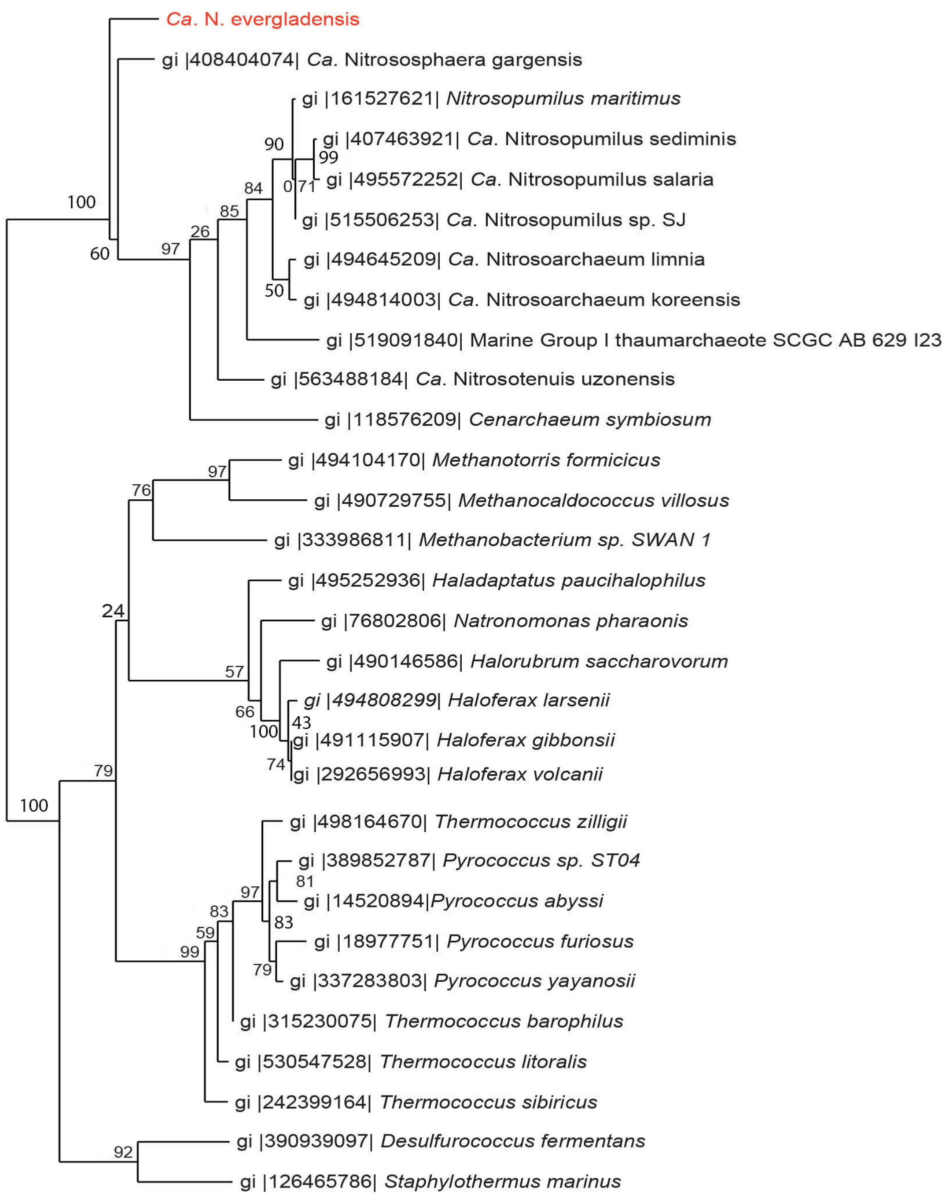

Figure S9. A phylogenetic tree of archaeal pelota gene homologs. Amino-acid sequences of pelota were randomly selected from the National Center for Biotechnology Information databases. The multiple sequence alignment of the amino-acid sequences was used for building maximum-likelihood trees.

Table S1. Protein coding sequences of central carbon, nitrogen, lipid metabolism and genes involved in the stress response of the archaeon

File S2

Table S2. Transporters encoded in *Ca*. N. evergladensis genome

File S2

Table S3. Information processing machinery of *Ca*. N. evergladensis

File S2

Table S4. Protein coding sequences (COGs and TIGRfams) present only in the AOA group I.1b

File S2

Table S5. Coding sequences present only in *Ca*. N. evergladensis genome but missing from the genome of *Ca*. N. gargensis

File S2

Table A. Comparison of *Ca.* N. evergladensis with other AOA genomes that are available in the public databases. CDS were compared at amino-acid identity ≥ 35%

Ammonia-oxidizing archaeon	Group	Total number of CDS	Number of shared CDS with *Ca.* N. evergladensis	Nucleotide sequence identity, %	
				16S rRNA	*amo*A
Ca. Nitrososphaera gargensis Ga9.2	I.1b	3562	2232	97	87
Nitrosopumilus maritimus SCM1	I.1a	1797	1187	85	73
Ca. Nitrosopumilus sediminis AR2	I.1a	1974	1233	85	72
Cenarchaeum symbiosum A	I.1a	2017	1115	85	74
Ca. Nitrosoarchaeum limnia SFB1	I.1a	2038	1252	85	73
Ca. Nitrosopumilus koreensis AR1	I.1a	1890	1203	85	71

Table B. Sequencing reports fro the Ion Torrent platform and Pacific Biosciences platform

**Technology**	**Number of runs/cells**	**Number of reads***	**Average read length, bp***	**Maximum read length, bp***	**Minimum read length, bp***	**Genome coverage**
Pacific Biosciences	9 SMART cells	197,138	4,117	21,581	500	~179X
Ion Torrent	Ion 318^{TM} Chip	2,389,864	241	477	80	~127X

*After quality trim

Table C. Comparative results of different assembly methods and sequencing technologies

**Assembler**	**Technology**	**G/C content, %**	**Total bp**	**Identity to *N. gargensis* genome**	**N50**	**Max. contig size, bp**	**Min. contig size, bp**	**Number of contigs**	**Greengenes Find ORF tool**
Celera	PacBio	50.14	2954373	40%	2954373	2954373	2954373	1	7454
Mira	PacBio	50.88	3372381	40%	716163	1232912	15072	21	8848
Mira	Ion Torrent	49.83	2814846	39%	222664	418142	7378	24	7162
IDBA-UD	Ion Torrent	50.27	2317515	32%	11511	41248	5021	212	5810

Methods

Quantitative PCR assays

Quantitative PCR (qPCR) for archaeal was performed in triplicate in the Mx3000P real-time PCR Thermal Cycler (Stratagene, La Jolla, CA, USA). SYBR Green assay was carried out in 25 µL PCR mixture volume consisting of 20 ng of DNA template, 2X QuantiTect® SYBR® Green Master Mix (Qiagen, Valencia, CA, USA), and 100 µM of each primer. The archaeal *amoA* gene fragment was amplified using the primer set Arch-amoAf (5'-STAATGGTCTGGCTTAGACG-3') and Arch-amoAr (5'-GCGGCCATCCATCTGTATGT- 3') (Francis *et al*., 2005). PCR cycling was performed as follows: one initial denaturation step at 95°C for 15 min followed by 40 cycles of denaturation at 94°C for 30 s, annealing at 56°C for 1 min, and extension at 72°C for 1 min with an efficiency of 110% and R^2 value of 0.987. Standard curves for interpretation of Ct values were generated with serial dilutions of a known copy number of the gene. Ct (threshold cycle) is the cycle number at which the fluorescence emission crosses a threshold within the logarithmic increase phase. For AOA *amoA* quantification, a linearized plasmid (pCR4-TOPO, Invitrogen, Carlsbad, CA, USA) containing a fragment of 2093 bp of amoBCA of *Nitrosopumilus maritimus* (Stahl lab, University of Washington) was used to create a standard curve. Results were expressed in relative abundance, $\log_{10}$ of gene copies per nanogram of DNA.

Trimming and sequence filtering of PacBio sequences

Raw data from PacBio was initially processed for finding the highest scoring local alignments among reads with BLASR from SMRT Analysis portal 1.4 (http://www.pacbiodevnet.com/SMRT-Analysis/Algorithms/BLASR). The sequences were also filtered by length before assembly, using 8859 nucleotides as cutoff value. Both filtering steps resulted in 8602.5 average read size (6964 reads, N50=9684) with 0.858 read quality. This final filtering by size was crucial to obtain the present genome from the assembly with Celera. High number of short reads dramatically increases computing requirements and may also result in a worse quality assembly due to the excess of error reads. The initial sequencing report data with BLASR filtering is available in **Table B**.

Sequence assembly

In order to verify the presence of error in the present genome assembly, we compared the results of different *de novo* assembly tools and sequencing technologies. A detailed comparison among the assembly results obtained from different methods and technologies is in **Table C**.

IonTorrent

The trimmed/filtered IonTorrent reads were assembled using the *de novo* genomic assembly tools Mira 3.9 (Chevreux et al., 1999) and IDBA-UD (Peng et al., 2012). IDBA-UD algorithm is based on the de Bruijn graph approach for assembling reads from single-cell sequencing or metagenomic sequencing technologies with uneven sequencing depths. Mira takes advantage of additional available information such as low confidence regions, quality values or repetitive region tags in order to improve the assembly procedure. In both *de novo* assemblers, we used the parameters optimized for the present reads, with non-uniform read distribution, accurate assembly options and no trace information.

PacBio

After reads filtering/trimming PacBio reads, we used Celera tool from SMRT portal for assembly (http://www.pacbiodevnet.com/ SMRT-Analysis/Software/SMRT-Pipe). Celera is a tool for scalable genome assembly of PacBio long reads. The default settings for PacBio reads were used in Celera assembly run. In addition, we used MIRA assembler as an alternative method in order to compare its generated contigs to Celera results. The default options were used in MIRA assembler for PacBio reads.

Genome finishing and scaffolding

The final PacBio contig (present genome) was also filtered with Quiver (Chin *et al*., 2013), a highly accurate consensus and variant caller that can generate 99.99% accurate consensus sequences using local realignment and the full range of quality scores associated with PacBio reads (We obtained a consensus concordance of 99.9945 for the present genome).

Assembly Verification

After the assembly procedure, we compared the contigs generated by both sequencing technologies (PacBio and Ion Torrent). We used Vista (Frazer *et al*., 2004) and Mauve (Darling et al., 2004) genomic analysis

tools to align the final contig generated by Celera to all contigs generated by Mira. Vista results shows 99% of conserved nucleotides between Celera (PacBio) and Mira (IonTorrent) contigs and all assemblers have shown similar GC content (**Table C**).

Annotation

Two-component systems annotation

We used Conserved Domain Search tool from NCBI (Gibney, Baxevanis, 2011). We compared and merged the results from two different databases: TIGRfam and Conserved Domain Database (CDD) (Scott et al., 2011). We used 0.01 as *e-value* cut-off for both databases. We selected the lowest *e-value* unique hits with high coverage (≥75%). In some the CDD results, multiple domain hits of different types of two-component systems matched to the same locus tag. In those cases, we maintained the multiple domain hits with similar e-values. This problem was not observed in TIGRfam database search.

Phylogenetic analyses

The nucleotide and amino acid sequences for phylogenetic reconstruction were obtained from NCBI databases (Gibney, Baxevanis, 2011; Benson *et al*., 2012). The selected nucleotide sequences of 16S rRNA and predicted amino acid sequences of AMO were aligned using the multiple sequence analysis tool MUSCLE 3.8.31 (Edgar, 2004). The multiple sequence alignment of 16S rRNA was filtered using GLBLOCKS for selecting conserved sites and remove poorly aligned regions (Talavera, Castresana, 2004). Conserved blocks were selected using the following criteria: at least 8 sites per conserved block length, with up to 4 contiguous nonconserved regions, present in at least 50% of the aligned sequences.

Maximum likelihood trees were built using the phylogenetic tree reconstruction tool PhyML 3.0 (Criscuolo, 2011), using the default parameters and 100 bootstraps for 16S sequences and JTT model, 1000 bootstraps, and best of NNI and SPR search operations for amino acid sequences of AMO. The phylogenetic trees were visualized and exported using Archaeopteryx 0.9809 tool (Han, Zmasek, 2009).

References

Francis CA, Roberts KJ, Beman JM, Santoro AE, Oakley BB (2005) Ubiquity and diversity of ammonia-oxidizing archaea in water columns and sediments of the ocean. Proc Natl Acad Sci U S A 102: 14683-8.

Chevreux B, Wetter T, Suhai S (1999) Genome Sequence Assembly Using Trace Signals and Additional Sequence Information. In: German Conference on Bioinformatics 1999: 45-56.

Peng Y, Leung HC, Yiu S-M, Chin FY (2012) IDBA-UD: a de novo assembler for single-cell and metagenomic sequencing data with highly uneven depth. Bioinformatics 28: 1420-1428.

Chin CS, Alexander DH, Marks P, Klammer AA, Drake J, et al. (2013) Nonhybrid, finished microbial genome assemblies from long-read SMRT sequencing data. Nat Methods 10: 563-9.

Frazer KA, Pachter L, Poliakov A, Rubin EM, Dubchak I (2004) VISTA: computational tools for comparative genomics. Nucleic Acids Res 32: W273-279.

Darling AC, Mau B, Blattner FR, Perna NT (2004) Mauve: multiple alignment of conserved genomic sequence with rearrangements. Genome Res 14: 1394-1403.

Gibney G, Baxevanis AD (2011) Searching NCBI databases using Entrez. Curr Protoc Bioinformatics Chapter 1: Unit 6.10.

Marchler-Bauer A, Lu S, Anderson JB, Chitsaz F, Derbyshire MK, et al. (2011) CDD: a Conserved Domain Database for the functional annotation of proteins. Nucleic Acids Res 39: D225-229.

Benson DA, Karsch-Mizrachi I, Clark K, Lipman DJ, Ostell J, Sayers EW (2012) GenBank. Nucleic Acids 40: D48-53.

Edgar RC (2004) MUSCLE: multiple sequence alignment with high accuracy and high throughput. Nucleic Acids Res 32: 1792-1797.

Talavera G, Castresana J (2007) Improvement of phylogenies after removing divergent and ambiguously aligned blocks from protein sequence alignments. Syst Biol 56: 564-577.

Criscuolo A (2011) morePhyML: improving the phylogenetic tree space exploration with PhyML 3. Mol Phylogenet Evol 61: 944-948.

Han MV, Zmasek CM (2009) phyloXML: XML for evolutionary biology and comparative genomics. BMC Bioinformatics 10: 356.
